# Promoter-dependent nuclear RNA degradation ensures cell cycle-specific gene expression

**DOI:** 10.1038/s42003-019-0441-3

**Published:** 2019-06-17

**Authors:** Mathieu Catala, Sherif Abou Elela

**Affiliations:** Département de microbiologie et d’infectiologie, Faculté de médecine et des sciences de la santé, Université de Sherbrooke, Sherbrooke, QC J1E 4K8 Canada

**Keywords:** RNA decay, Mitosis

## Abstract

Cell cycle progression depends on phase-specific gene expression. Here we show that the nuclear RNA degradation machinery plays a lead role in promoting cell cycle-dependent gene expression by triggering promoter-dependent co-transcriptional RNA degradation. Single molecule quantification of RNA abundance in different phases of the cell cycle indicates that relative curtailment of gene expression in certain phases is attained even when transcription is not completely inhibited. When nuclear ribonucleases are deleted, transcription of the *Saccharomyces cerevisiae* G1-specific axial budding gene *AXL2* is detected throughout the cell cycle and its phase-specific expression is lost. Promoter replacement abolished cell cycle-dependent RNA degradation and rendered the RNA insensitive to the deletion of nuclear ribonucleases. Together the data reveal a model of gene regulation whereby RNA abundance is controlled by promoter-dependent induction of RNA degradation.

## Abstract (web summary)

Catala and Abou Elela show that the nuclear RNA degradation is executed in a promoter-specific manner, ensuring cell cycle-dependent gene expression. This study illustrates that local gene expression can be temporarily regulated without affecting overall transcription.

## Introduction

Cells proliferate through a well-coordinated cycle ensuring the replication and redistribution of all cellular components^1–3^. This cell cycle requires the synthesis of a panel of proteins required for replication, many of which are expressed only when they are needed. Consequently, progression through the cell cycle is accompanied by dramatic reorganization of gene expression that is often referred to as cell cycle-regulated transcription^3^. 20% of yeast genes are expressed in a cell cycle-dependent manner, guided by the activation of transcription factors and cycling genes^4^. These waves of expression are often divided into four events that take place in G1, S, G2/M, and in the transition between the M and G1 phases. Genes associated with these waves are classified into clusters based on expression patterns and promoter motifs that promote cell cycle-regulated transcription^3^. Furthermore, shared common promoter elements are co-regulated by specific subsets of transcription factors, like the G1/S SCB-binding factor and MCB-binding factor^5,6^. These promoters can be used experimentally to induce phase-specific expression of reporter proteins^7,8^.

More recently, cell cycle changes have been associated with changes in RNA stability^9^. Metabolic RNA labeling and dynamic transcriptome analysis identified hundreds of genes in budding yeast with periodic changes in, transcription and mRNA degradation, rates^9–13^. In general RNA degradation follows peaks of mRNA synthesis at defined times during the cell cycle. Strikingly, it was shown that the promoters of two mitotic genes (*SWI5* and *CLB2)* trigger cell cycle-dependent RNA degradation in the cytoplasm^14^. In these two cases, it was proposed that RNA is marked for decay by factors loaded on the RNA during transcription, leading to cytoplasmic degradation at the end of the cell cycle. However, it is not clear how the factors are recruited to the promoter, nor how the RNA is degraded in the cytoplasm. It is also unclear if co-transcriptional RNA degradation in the nucleus contributes to cell cycle repression. A few examples of targeted nuclear RNA degradation have been linked to nutritional changes and other stresses^15–17^. However, it remains unclear generally how nuclear RNA degradation contributes to transcriptional regulation or how it regulates specific cell cycle genes.

Most biochemical assays provide estimates of average RNA amounts in a population of cells but cannot distinguish between nuclear and cytoplasmic RNA degradation. In such assays it is difficult to determine the location and timing of gene expression and the resolution of the expression cycle is often blurred by stochastic differences between cells. In this study, we monitored the synthesis and degradation of the phase-specific axial budding gene *AXL2* during the cell cycle using single molecule analysis to determine the location, timing, and mechanism of phase-specific gene expression. Like many cell cycle genes *AXL2* is thought to be expressed in the G1 phase of the cell cycle in a promoter-dependent manner^18,19^. However, in vitro studies suggested that this gene might also be regulated post-transcriptionally^20^. Here, we demonstrate that despite the sharp decline of *AXL2* transcripts at the end of G1, it is transcribed albeit at a lower level throughout the cell cycle; live cell analysis and chromatin immunoprecipitation of RNA polymerase II confirmed that transcription of *AXL2* continues in the S and G2/M phases of the cell cycle. Strikingly, deletion of nuclear ribonucleases completely blocked the characteristic periodicity of mature *AXL2* mRNA levels. Ribonucleases were associated with the chromatin linking the RNA degradation machinery to transcription. Consistently, substitution of the *AXL2* promoter impaired the cycling of gene expression. Together our data reveal a model of gene expression, in which cell cycle expression is achieved through promoter-dependent RNA degradation.

## Results

### RNA decay drives the cell cycle expression of *AXL2*

**Fig. 1:**
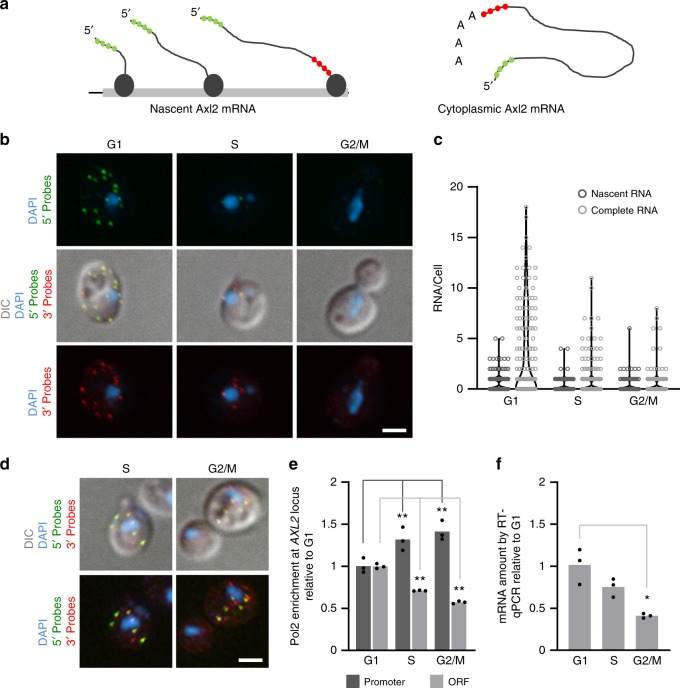
Single mRNA molecules analysis reveals synthesis of Axl2 mRNA across the cell cycle. **a** Schematic representation of the probes used to detect *AXL2* mRNA in the fluorescence in situ hybridization (FISH) assay shown in (**b**). Probes complementary to the 5′ end of the target are shown in green and those to the 3′ end are red. **b** Monitoring *AXL2* expression through the cell cycle using single cell FISH assay. The RNA was detected using the probes illustrated in (**a**) and the nuclei stained with DAPI (blue). The cell cycle phases were determined using the bud size and nucleus position. The white bar equals 2 µm. **c** *AXL2* expression is downregulated during S and G2/M. Nascent mRNAs (dark gray) are defined as RNAs detected using the 5′ probe in the nucleus, while complete cytoplasmic mRNAs (light gray) are detected by both 5′ and 3′ probes in the cytoplasm. The number of RNAs per cell is shown in the violin plot for 150 cells analyzed per phase. **d** Examples of cells transcribing Axl2 mRNA in the S and G2/M. RNA was detected as described in (**b**). **e** Cells were synchronized in G1, S, or G2/M and the enrichment of RNAPII at the *AXL2* locus was analyzed by ChIP and normalized to the G1 enrichment. **f** The amount of Axl2 mRNA from the synchronized cultures used in (**e**) was determined using qRT-PCR and presented relative to the amount detected in G1. The  bar graphs represent the average of 3 independent biological replicates and the value of each replicate is shown as dot. (*p < 0.05 and **p < 0.01 by two-tailed paired *t* test)

To determine the regulatory events controlling the expression of cell cycle-associated genes, we monitored the life cycle of the axial budding gene *AXL2* using single-molecule fluorescence in situ hybridization (smFISH)^21^. *AXL2* expression is required in the G1 phase of the cell cycle, and defects in its expression are easily scored by changes in budding patterns^18^. The Axl2 mRNA was detected using two independent sets of single labeled 20 oligodeoxynucleotides probes (Fig. 1a). The first probe set hybridizes to the 5′ end of the mRNA, while the other hybridizes to the 3′ end. Signals generated by the 5′ end probe at the transcription site in the nucleus identify nascent RNA, while complete or mature RNA, which is mostly cytoplasmic is identified by signals generated from both 5′ and 3′ end probes. To determine the number of probes sufficient for accurate RNA detection we compared the signal intensity and the number of RNAs detected by 12- and 24-probe sets. The results indicated that while a set of 24 probes resulted in higher signal intensity indicating a higher number of RNAs detected, the use of 12 probes provided a narrower peak and less inter-experiment variation without changing the overall conclusion (Supplementary Fig. 1). Accordingly, we used the 12-probe set for all subsequent experiments comparing either nascent (nuclear) or complete (mostly cytoplasmic) RNA in different conditions and phases of the cell cycle. We also confirmed the accuracy of nuclear targeting of the transcripts by comparing compressed 2D- with uncompressed 3D images, as 2D stacking of the images, which simplify quantification, do not result in more than 10% inaccuracies (Supplementary Fig. 2).

Examining the expression pattern of Axl2 during the cell cycle using either the 5′ or the 3′ end probe reproduced the established expression pattern of Axl2 mRNA, which peaks in G1 and is repressed in later phases (Fig. 1b)^18^. We next analyzed the Axl2 expression dynamics quantitatively as our technique allows quantification of the number of nascent RNAs (hybridizing to the 5′ end probe only in the nucleus) and completed RNAs (hybridizing to both 5′ and 3′ end probes in the cytoplasm) per cell. Both nascent and full-length RNA decreased as the cells exited from the G1 phase of the cell cycle (Fig. 1c and Supplementary Fig. 3). On average 4 RNA molecules per cell were detected in G1, while only 1 molecule per cell was detected in S phase. Together these data illustrate the overall accuracy of the Axl2 mRNA targeted probes. We also found the amount of Axl2 mRNA varies depending on the cell size (Supplementary Fig. 3c). In general, more Axl2 mRNA was detected in larger G1 cells. In all cases RNA peaked in G1 and gradually dipped over S phase to a minimum in G2/M. However, cytoplasmic and nascent RNA did not decrease at the same rate; full length RNA decreased by 6-fold, whereas nascent RNA decreased only 3-fold (Fig. 1c). Notably, RNA was detected in the nucleus in all phases of the cell cycle suggesting that transcription continues throughout the cell cycle (Fig. 1d). To verify the continuing transcription of the *AXL2* gene beyond the G1 phase of the cell cycle, we synchronized cultures and compared the association of RNA polymerase (RNAPII) at the *AXL2* locus to accumulation of its RNA.

As indicated in Fig. 1e, the association of RNAPII with the promoter of *AXL2*, did not decrease but instead, increased in the S and G2/M phases. On the other hand, the association of RNAPII with the *AXL2* open reading-frame was modestly reduced but continued to be detected in the S and G2/M phases. This data clearly indicate that Axl2 mRNA transcription continues beyond G1. Consistently, monitoring the accumulation of Axl2 mRNA in the same synchronized culture by RT-qPCR once again indicated that Axl2 mRNA abundance decreases but remains detectable in S and G2/M (Fig. 1f) Together these results clearly indicate that the cycling of *AXL2* expression is not produced by strict repression and induction of transcription.

**Fig. 2:**
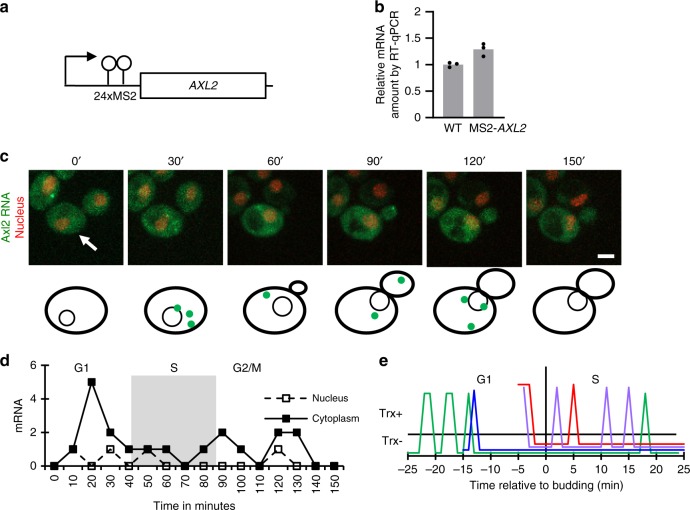
Live cell analysis of Axl2 mRNA expression throughout the cell cycle. **a** Schematic representation of the tagged *AXL2* gene used in live cell assays. The 24 MS2-coat-protein binding sites inserted in *AXL2* 5′ UTR are indicted as hairpins. The empty box and arrow represent the *AXL2* gene and its transcription start site, respectively. **b** Impact of the MS2 tag on the abundance of Axl2 mRNA. The relative abundance of tagged and untagged mRNA was determined by qRT-PCR. The bar graphs represent the average of 3 independent biological replicates and the value of each replicate is shown as dot. **c** Live cell imaging of Axl2 mRNA. The MS2-tagged Axl2 mRNA was visualized, through the binding of MCP-GFP to MS2, and the images taken every 10 min are shown (for complete image sequence see Supplementary Fig. 5). The nucleus is visualized in red by the expression of td-tomato-NLS. The cell monitored in this study is indicated by arrow. The white bar equals 2 µm. The position of the RNA detected in different frames is shown at the bottom. The specificity of the spots was examined in Supplementary Fig. 4b. **d** Quantification of Axl2 mRNA detected in live cells. The number of RNA per cell detected every 10 min was calculated as described in the “Methods” section and plotted relative to time in minutes. The different phases of the cell cycle as function of the bud size are indicated on top. **e** Detecting transcriptional pulses of *AXL2* in the G1 and S phases of the cell cycle. The RNA was captured every minute and plotted relative to the budding time. Budding start time (0) is indicated by vertical line. Each colored line represents data from a total of 4 cells. Trx+ and Trx− indicated transcription and no transcription detected, respectively

To monitor the dynamics of *AXL2* expression, we examined the accumulation of Axl2 mRNA in real-time (Fig. 2 and Supplementary Figs. 4 and 5). We did this by inserting repeats of the MS2 coat protein binding site into the 5′UTR of the gene (Fig. 2a), which then acted as a reporter of the mRNA by attracting a coat protein-EGFP fusion in live cells^22,23^. We have used the tag version and location previously certified for the detection of transcription and nascent RNA^22^. Tagging Axl2 mRNA slightly increased its abundance but did affect its expression cycle (Fig. 2b, c). Indeed, the overall number of spots detected by the tagged version was similar to those detected by the untagged version using FISH (compare, Figs. 1b and 2c). We monitored Axl2 mRNA every 10 min across the cell cycle and every minute across the G1/S transition. Interestingly, de novo transcription was detected in the nucleus at all phases of the cell cycle and was not completely repressed in S and G2/M (Fig. 2c). On the other hand, cytoplasmic RNA was clearly reduced in S and G2/M. Interestingly, while there were only 1 and 0.6 Axl2 RNAs per cell in S and G2/M phases respectively on average, transient pulses of RNA expression were detected in live cells in these phases (Fig. 2d). This indicates that RNA is both made and degraded during S and G2/M. It should be noted that since the pictures were taken every 10 min and the transcription pulses take ~2 min followed by the export or degradation of the RNA many RNA transcription events may escape detection^23^. Indeed, acquiring images every minute reveals similar number of transcription pulses in the G1 and S phases (Fig. 2e). This clearly indicates once again that the rapid decrease in Axl2 mRNA amounts is not only due to transcription repression. We conclude that the phase-specific expression of *AXL2* is not only created by peak transcription in G1 but through accelerated RNA decay in S phase.

### Nuclear ribonucleases promote *AXL2* cell cycle-dependent repression

**Fig. 3:**
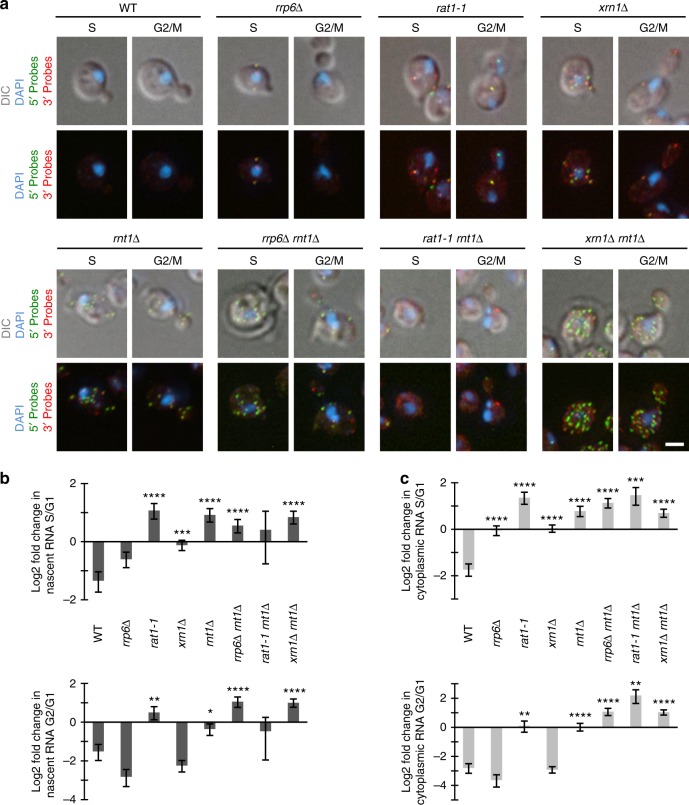
Nuclear ribonucleases are essential for the cell cycle-dependent repression of *AXL2*. **a** smFISH analysis of cells with the various ribonuclease mutations shown. The expression of Axl2 mRNA was monitored in wild type cells (WT) or cells with single (*rrp6∆*, *rat1-1*, *rnt1∆*, and xrn1∆) or double (*rrp6∆ rnt1∆*, *rat1-1 rnt1∆, xrn1∆*, *and xrn1∆ rnt1∆*) mutations in different nuclear or nuclear/cytoplasmic ribonucleases. FISH analysis was performed as described in Fig. 1 and examples of cells in the S and G2/M phases, when *AXL2* is normally repressed, are shown. The white bar equals 2 µm. **b** Quantification of ribonuclease-dependent changes in nascent RNA levels (5′ end probe) in S (top panel) or G2/M (bottom panel). Nascent RNA detected in the nucleus was quantified in different phases and the Log2 change between S or G2/M relative to G1 is plotted for each mutant strain. **c** Effect of ribonuclease mutations on the abundance of cytoplasmic Axl2 mRNA. The total amount of full-length RNA detected in different phases was quantified and the Log2 change between the S (top panel) or G2/M (bottom panel) relative to G1 is plotted for each mutant strain. All error bars represent the uncertainties calculated from the standard errors of the mean (*p < 0.05, **p < 0.01, ***p < 0.001, ****p < 0.0001 by two-tailed unpaired *t* test with Welch’s correction)

The data above indicate that at the G1/S transition there is a discrepancy between the decrease in number of Axl2 mRNAs in the nucleus and its decrease in transcription, suggesting increased nuclear RNA decay. To evaluate the contribution of nuclear RNA degradation to cell cycle-dependent expression, we examined the effect of abolishing the activity of different nuclear ribonucleases on *AXL2* expression cycle. We deleted or inactivated 4 ribonucleases: three exoribonucleases (Rrp6p, Rat1p, and Xrn1p) and one endoribonuclease (Rnt1p). Rrp6p is a component of the nuclear exosome implicated in the 3′–5′ end degradation of non-polyadenylated RNA with free 3′ OH^24^. On the other hand, Rat1p is a 5′–3′ exoribonuclease that degrades uncapped RNA with 5′ monophosphate involved in transcription termination^25^. Unlike Rrp6p and Rat1p that are exclusively nuclear, Xrn1p is a mostly cytoplasmic paralog of Rat1p, but it has also been linked to nuclear RNA degradation^26^. Finally, Rnt1p is a nuclear endoribonuclease that exits from the nucleolus to the nucleoplasm at the end of the G1 phase of the cell cycle and cleaves RNA with conserved NGNN stem loop structures including one in Axl2 mRNA^20^. Mutating each of the 4 ribonucleases individually increased the abundance of both nascent and complete RNA in S phases when compared to that detected in G1 or when compared to the wild type RNA in the same phase (Fig. 3a). Similarly, certain ribonuclease mutations (e.g., *rat1-1* and *rnt1∆*) increased the ratio of the RNA in G2/M phase compared to G1 (Fig. 3b). However, only *RNT1* deletion increased cytoplasmic Axl2 mRNA in the G2/M phase compared to wild type while the detected RNA remained stable at around 0.2–0.5 copies per cell in the other ribonuclease deletions (Supplementary Fig. 6a). Surprisingly, *XRN1* deletion not only altered the amount of complete Axl2 mRNA in the cytoplasm but it also increased nascent RNA in the nucleus in G1 and S (Fig. 3b, c). This suggests that Xrn1p directly or indirectly contributes to Axl2 degradation in the nucleus. Double and triple deletions or mutations of exoribonucleases (e.g., *RRP6* and *XRN1*) increased the effect of the endoribonuclease *RNT1* in G2/M but not in S phase (Supplementary Fig. 6b). Therefore, RNA that is not cleaved by Rnt1p in S phase can be degraded by the other exoribonucleases later in the cell cycle. The other nuclear ribonucleases may also complete the degradation of RNA fragments generated by Rnt1p cleavage. Together these observations suggest that nuclear ribonucleases work together to ensure the rapid degradation of Axl2 mRNA in S phase. Strikingly, double deletion of *RNT1* and *XRN1* completely abolished the periodic expression of *AXL2* resulting in the production of increasing amounts of RNA as the cells grow (Supplementary Fig. 6a, b). We conclude that nuclear ribonucleases are required for the cell cycle-dependent repression of *AXL2*.

### Rnt1p is required for *AXL2* cell cycle-dependent repression

**Fig. 4:**
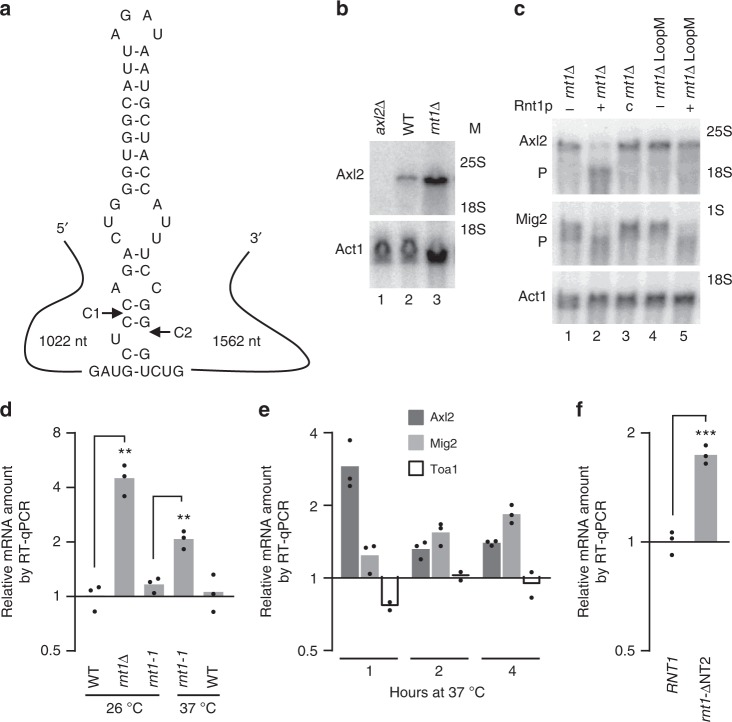
Axl2 mRNA is directly cleaved by the nuclear endoribonuclease Rnt1p. **a** Structure of Rnt1p nuclease-target site in Axl2 mRNA. Cleavage sites C1 and C2 are indicated by arrows and the size of the flanking sequence is shown on either side. **b** *RNT1* deletion increases the abundance of Axl2 mRNA. Axl2 mRNA was detected by northern blot in wild-type (WT) or *rnt1∆*. cells. Cells lacking *AXL2* (*axl2∆*) and Act1 mRNA are the negative and loading controls. The position of the rRNA size marker is indicated on the right. **c** In vitro cleavage assay of Axl2 mRNA. Total RNA extracted from *rnt1∆* cells (*rnt1∆)* or *rnt1∆* cells expressing Axl2 mRNA with mutated cleavage site (*rnt1*∆ LoopM) was incubated alone (−), with recombinant Rnt1p (+), or with catalytically inactive (*rnt1-*D245R) mutant of Rnt1p (**c**). The RNA was detected using probes against Axl2, Rnt1p substrate Mig2, or Act1 mRNA as negative control. The position of the uncleaved RNA and the 3′ cleavage product (P) is indicated on the left. **d** Short-term inactivation of Rnt1p increases the abundance of Axl2 mRNA. Axl2 mRNA was quantified by qRT-PCR in WT, *rnt1∆* or mutated thermosensitive *RNT1* (*rnt1-1*) cells grown at the permissive (26 °C) or restrictive temperature (37 °C). **e** Axl2 mRNA mimics the expression kinetics of Rnt1p substrates. The abundance of Axl2 and Rnt1p substrate Mig2 and unrelated Toa1 mRNA was examined after shifting to the restrictive temperature (37 °C). Axl2 and Mig2 mRNAs were analyzed in 3 N while Toa1 mRNA was analyzed in 2 N. **f** Rnt1p exit from the nucleolus to the nucleoplasm is required for the repression of *AXL2*. The abundance of Axl2 mRNA was determined in cells expressing wild type Rnt1p (*RNT1*) or a mutated version that accumulates in the nucleolus (*rnt1-∆*NT2). The bar graphs represent the average of 2-3 independent biological replicates and the value of each replicate is shown as dot. (**p < 0.01, ***p < 0.001 by two-tailed unpaired *t* test)

We next focused on Rnt1p as it is the only ribonuclease of the four tested with well-defined substrate selectivity^27^, a deletion phenotype of net increase in the Axl2 mRNA (Supplementary Fig. 6a), and previously shown Axl2 mRNA cleavage activity in vitro^20^. We thus studied if and how Rnt1p endoribonuclease represses *AXL2* during the cell cycle. To test this, we first re-evaluated the impact of *RNT1* deletion on Axl2 mRNA in vivo and the specificity of Rnt1p’s cleavage of Axl2 mRNA in vitro. Total RNA was extracted from wild type cells, cells lacking *RNT1* (*rnt1∆*) or cells expressing a mutation in the Axl2 mRNA stem-loop recognized by Rnt1p (*rnt1∆* LoopM, Fig. 4a). We then incubated the RNAs with, or without, recombinant Rnt1p. As expected, deletion of *RNT1* increased the overall abundance of Axl2 mRNA, confirming the results obtained by smFISH (Fig. 4b, c). Recombinant enzyme generated the previously seen cleavage product (P) 14–16 nucleotides from the loop^20^. The cleavage was dependent on both the presence of the site that Rnt1p cleaves (*rnt1∆* LoopM+) and the catalytic activity of Rnt1p (*rnt1∆* c) (Fig. 4c). This clearly illustrates the accuracy and specificity of Rnt1p’s cleavage of Axl2 mRNA.

The stem-loop mutation (LoopM) that blocks Axl2 mRNA cleavage in vitro (Fig. 4c) also increased the abundance of Axl2 mRNA, but only in the absence of the exoribonucleases Xrn1p and Rrp6p (Supplementary Fig. 7a). Deleting *RNT1* in the strains carrying the stem-loop and exoribonuclease mutations (*rnt1∆ rrp6∆ xrn1∆* LoopM) did not increase the abundance of nascent or full-length RNA in S phase (Supplementary Fig. 7b). In contrast, deletion of *RNT1* increases the abundance of Axl2 mRNA in the G2/M phase even when the loop is mutated. Therefore the site that Rnt1p cleaves is required for Axl2 repression in S phase and Rnt1p is required for maintaining the repression during G2/M. The reason why Rnt1p loop mutation by itself did not increase RNA abundance is likely because of degradation by other exoribonucleases that are otherwise inactivated by *RNT1* deletion, as previously shown for other substrates of Rnt1p^15^. Notably, the catalytically inactive Rnt1p mutant that nevertheless preserves RNA binding, increased *AXL2 m*RNA abundance and partially inhibited its cycling, clearly indicating Axl2 cleavage is required for its repression (Supplementary Fig. 7c).

To differentiate between direct and secondary effects of *RNT1* deletion, we examined the impact of short-term inactivation of Rnt1p activity on the abundance of Axl2 mRNA and compared it to the effect of *RNT1* deletion by qRT-PCR. As expected, the deletion of *RNT1* (*rnt1*∆) had the most impact on Axl2, increasing its mRNA 4-fold (Fig. 4d). Strikingly, the increase in Axl2 RNA was also observed shortly after the inactivation of a temperature-sensitive allele of *RNT1* (*rnt1-1*)^28^, consistent with direct cleavage by Rnt1p (Fig. 4d). Indeed, the increase in Axl2 mRNA was observed after only 1 h of shift to the restrictive temperature, at the same time as change in the abundance of the known substrate Mig2 mRNA^16^ is observed and before any increase in an unrelated mRNA, Toa1 (Fig. 4e). Together these data confirm the direct link between Rnt1p catalytic activity and the increase in Axl2 mRNA abundance. Since Rnt1p accumulates exclusively in the nucleus its deletion would be expected to increase the number of RNAs leaving the nucleus without affecting the overall half-life of the majority of the RNA that accumulates in the cytoplasm. Accordingly, there was no significant difference between the half-life of Axl2 RNA extracted from wild-type and *rnt1*∆ cells (Supplementary Fig. 8). This further confirms the specificity of Rnt1p, on the nascent Axl2 transcripts, observed above by smFISH. To further prove the specificity of Rnt1p effect on Axl2 mRNA we examined the impact of Rnt1p localization on the abundance of Axl2. Axl2 mRNA is produced in the nucleoplasm and Rnt1p exits from the nucleolus to the nucleoplasm in S phase. Therefore, blocking this exit of Rnt1p from the nucleolus should prevent the cleavage of Axl2 and increase it incrementally in S and G/2M. As indicated in Fig. 4f, deletion of the N-terminal domain of Rnt1p, which blocks the cell cycle-dependent exit of Rnt1p from the nucleolus^29^, increased Axl2 mRNA. We conclude that repression of *AXL2* requires Rnt1p to leave the nucleolus in S phase.

**Fig. 5:**
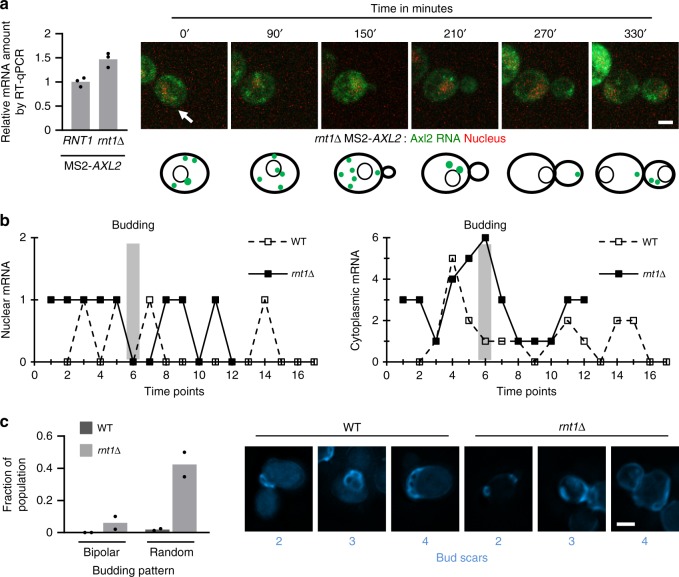
Rnt1p is required for the cycling and function of Axl2 mRNA. **a** Effect of *RNT1* deletion on *AXL2* expression during the cell cycle. The nucleus and RNA were visualized in *rnt1∆* cells in real time as described in Fig. 2c. The RNA was imaged every 30 min to capture the changes in the different stages of budding. The white arrow indicates the position of the budding cell of interest. The white bar equals 2 µm. Schematic of Axl2 mRNA expression is shown below. The abundance of tagged RNA in *RNT1* or *rnt1∆* strains was examined using qRT-PCR to confirm the tag neutrality and the average values from 3 independent biological replicates represented as bar graphs with individual replicates shown as dots (shown on left). **b** Comparison of the Axl2 mRNA expression cycle in wild type and *rnt1∆* cells. The graph presents the amount of RNA in the nucleus (left) or the cytoplasm (right) in the presence (WT) or absence (*rnt1∆*) of *RNT1*. Please note that the sampling time is different for each strain due to their different growth rates. Time-points are presented in order of acquisition and aligned relative to the time of budding. **c** Deletion of *RNT1* impairs Axl2p-dependent budding. The budding pattern was detected in the presence and the absence of *RNT1* by monitoring the bud scars using calcofluor staining. The fraction of the population with non-axial budding (i.e., bipolar or random) for 2 N with population samples totalizing 115 WT cells and 118 *rnt1∆* cells is reported. The bar graph and dots represent the average and individual values of the replicates (left panel). Examples of the scaring patterns as observed in cells with 2–4 scars are shown on the right. The white bar equals 2 µm

Live-cell analysis also indicated that *RNT1* deletion perturbs the cycling of Axl2 mRNA (Fig. 5a and Supplementary Fig. 5). The presence of the tag did not inhibit the effect of *RNT1* deletion as an increase in MS2 tagged Axl2 mRNA was still observed albeit to a lower extent upon deletion of *RNT1* in the tagged strain (Fig. 5a). Both nuclear and cytoplasmic RNA continued to accumulate before and after budding (Fig. 5b). However, it was not clear whether Rnt1p induced degradation of Axl2 mRNA led to changes in the abundance of Axl2 protein or affected Axl2 function. Therefore, we examined the impact of *RNT1* deletion on Axl2-dependent axial budding pattern. Cells were stained using the bud scar stain calcofluor and number and position of bud scars were compared between wild type and *rnt1∆* cells. As indicated in Fig. 5c, deletion of *RNT1* increased the number of random budding pattern normally associated with *AXL2* overexpression^18^. As expected, the defect in budding pattern was not exaggerated by multiple nuclease deletion confirming that the different ribonucleases affect budding through the same pathway (Supplementary Fig. 9). We conclude that nuclear mRNA degradation inhibits the production of Axl2 protein to maintain budding patterns.

### Rnt1p cotranscriptionally degrades Axl2 mRNA

**Fig. 6:**
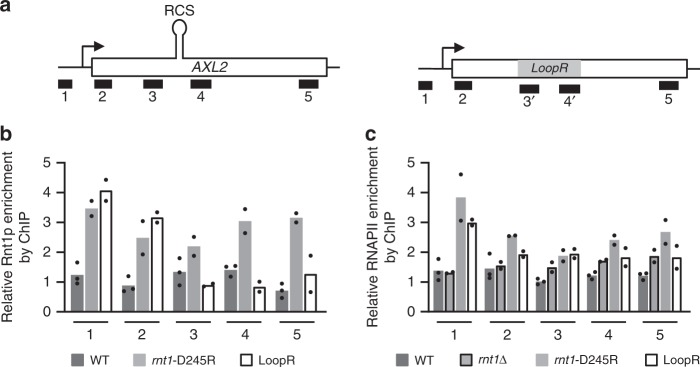
Rnt1p associates with both the promoter and RNA of *AXL2*. **a** Schematic representation of the *AXL2* locus depicting the position of the different fragments amplified by qPCR after chromatin immunoprecipitation. The position in the RNA of the Rnt1p cleavage site (RCS) is shown on top. The position of the loop sequence replacement is shown by a gray box. 3′ and 4′ indicate probe sets specific to the mutated sequence (LoopR). **b** Rnt1p associates with *AXL2* chromatin in both a promoter and RNA-cleavage site-dependent manner. Chromatin was immunoprecipitated using wild type cells (WT), cells expressing a catalytically impaired version of Rnt1p (*rnt1-D245R*) or cells expressing Axl2 mRNA lacking the sequence of the cleavage site (LoopR). The chromatin was precipitated using antibodies against Rnt1p^46^ and different fragments of *AXL2* were amplified using qPCR analysis using *AXL2* specific probes. The position of the different amplicons is illustrated in (**a**). **c** The deletion of *RNT1* increases RNAPII association with the *AXL2* locus. Chromatin was immunoprecipitated from the different cells described in (**b**) and *rnt1∆* with antibodies against RNAPII and the enrichment of the different *AXL2* fragments was evaluated using qPCR as described in (**b**). The enrichments were measured relative to a non-coding region of chrV. The bar graphs represent an average of 3 biological replicates for the wild type strains and 2 biological replicates for all other strains. The data points are shown in the form of dots

It was previously shown that Rnt1p cleaves its substrates cotranscriptionally^16,30,31^ explaining the rapid decrease in nascent *AXL2* transcripts upon the exit from G1. To evaluate this possibility, we examined the association of Rnt1p with the *AXL2* locus using chromatin immunoprecipitation (ChIP). As indicated in Fig. 6a, b, Rnt1p was modestly enriched in the promoter region and Rnt1p binding site but the enrichment was noticeably increased when the turn over of the enzyme is blocked (*rnt1*-D245R). Indeed, impairing the catalytic activity of Rnt1p increased by 3 folds the enzyme association with *AXL2* chromatin (Fig. 6b probes 1 and 2). Active enzyme rapidly turnovers decreasing the stability of its association with chromatin while the catalytically impaired version does not^32–34^. Replacement of the stem loop structure (LoopR) inhibited Rnt1p association with *AXL2* chromatin near the cleavage site, while increasing the interaction with promoter region (Fig. 6b probes 1, 3, and 4). Surprisingly, when Rnt1p binding site was replaced, Rnt1p association with *AXL2* promoter and the open reading-frame 5′ end increased dramatically. This suggests that Rnt1p associates with the chromatin of *AXL2* using two mechanisms: the first depends on the RNA binding site, which is linked to cleavage, and the other depends on the promoter sequence, perhaps through association with transcription factors.

To evaluate the contribution of Rnt1p to the transcription of *AXL2*, we monitored the impact of *RNT1* deletion on RNAPII occupancy by ChIP. As indicated in Fig. 6c, deletion of *RNT1* does not affect the overall level of RNAPII occupancy near the promoter and the 5′ end of the *AXL2* open reading-frame (Fig. 6c probes 1 and 2). Instead, the deletion increased RNAPII occupancy with the region surrounding the cleavage site in *AXL2* RNA, suggesting that cleavage may result in RNAPII release (Fig. 6c probes 3 and 4). This is consistent with the established role of Rnt1p in inducing transcription termination^30^. Therefore, it is the cleavage by Rnt1p, rather than its association with the chromatin, that decreases RNAPII occupancy. Indeed, inactivation of Rnt1p catalytic activity increases RNAPII association with *AXL2* RNA suggesting that, in the absence of RNA cleavage, association of Rnt1p with chromatin may lead to transcriptional pausing (Fig. 6b, c). The data is therefore consistent with the model whereby cleavage of RNA by Rnt1p initiates transcription termination by generating an entry point for the ribonuclease Rat1p, as previously shown for the *NPL3* gene^30,31,35^.

**Fig. 7:**
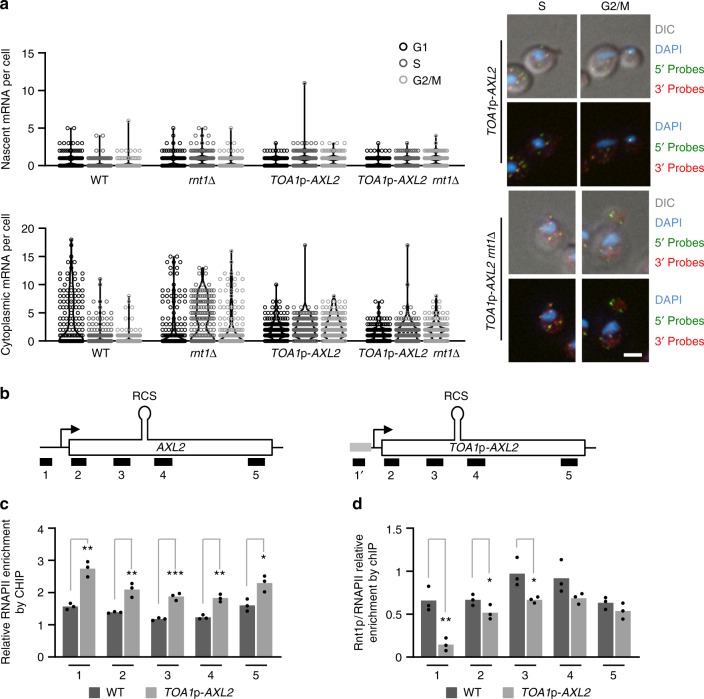
The cell cycle-dependent degradation of Axl2 mRNA is induced by its promoter. **a** The promoter of *AXL2* is required for Rnt1p-dependent repression of gene expression. The abundance of Axl2 mRNA was monitored in wild type or *rnt1*∆ cells expressing *AXL2* from its own promoter (WT and *rnt1∆*) or a heterologous promoter (*TOA*1p-*AXL2 and TOA1*p-*AXL2 rnt1*∆). The mRNA was quantified as described in Fig. 1c and is shown on the left while examples of cells captured in S and G2/M phases are shown on the right. White bar equals 2 µm. **b** Schematic representation of the *AXL2* locus depicting the position of the different fragments amplified by qPCR after chromatin immunoprecipitation. The position of the RNA target Rnt1p cleavage site (RCS) is shown at the top and the *TOA1* promoter is indicated by a gray box. The probe specific to the substituted sequence is indicated by 1′. **c** The promoter substitution increases the association of RNAPII with the *AXL2* locus. The RNAPII association pattern was examined in wild type cells (WT) or cells expressing *AXL2* from a heterologous promoter (*TOA*1p-*AXL2*) as described in Fig. 6c. **d** Substitution of the *AXL2* promoter inhibits the recruitment of Rnt1p to the *AXL2* locus. Rnt1p association with the *AXL2* locus, expressed from a wild-type or heterologous promoter, was examined as described in Fig. 6b with primers for amplicon 1 specific to the *AXL2* or *TOA1* promoter as required. Shown is the enrichment of Rnt1p relative to RNAPII. The bar graphs represent the average values obtained from 3 independent biological replicates shown as dots. Asterisks indicate significant difference between strains (*p < 0.05, **p < 0.01, ***p < 0.001 by two-tailed unpaired *t* test)

Since Rnt1p associates with chromatin near the promoter of *AXL2*, we examined the role of the promoter sequence on its association with Rnt1p and on *AXL2* expression. For this we used the promoter of the *TOA1* gene as it is expressed at similar levels to *AXL2* independently of the cell cycle^36–38^. Substitution of *AXL2* promoter with the unrelated *TOA1* promoter impaired the cycling of *AXL2* expression both in the presence and absence of Rnt1p. Remarkably, expression of *AXL2* from the *TOA1* promoter did not decrease in any phase of the cell cycle and the deletion of *RNT1* had no additional effect on the expression of the *TOA1*p-*AXL2* gene (Fig. 7a). A ChIP assay indicated that the substitution of the *AXL2* promoter inhibited the association of Rnt1p with the promoter region, but not its overall association with chromatin (Fig. 7b–d and Supplementary Fig. 10). This indicates that the promoter sequence helps recruit Rnt1p to the transcription site. Strikingly, promoter substitution also decreased the occupancy of Rnt1p at its cleavage site on *AXL2* RNA indicating once again that the promoter sequence is required for cleavage by Rnt1p (Fig. 7d). We conclude that *AXL2* expression is regulated by cell cycle-induced promoter-dependent RNA decay.

## Discussion

It is believed that modulation of gene expression is always linked to changes in transcription rates. For example, the cell cycle is often viewed as a periodic sequence of waves of transcription of phase-specific genes^3^. Here, we challenge this notion and demonstrate that the cell cycle may modulate gene expression of constitutively transcribed genes. We have shown that the transcription of the cell cycle-regulated gene *AXL2* continues despite the sharp decrease in the number of mature RNAs accumulating in S phase (Fig. 1 and Supplementary Fig. 3). The decrease in RNA depends on a network of nuclear ribonucleases, ensuring rapid and maintained repression of *AXL2* expression in S and G2/M (Fig. 3). Surprisingly, the cell cycle-dependent degradation of Axl2 mRNA depends on the specific promoter sequence (Fig. 7). A heterologous promoter impaired the recruitment of nuclear endoribonuclease Rnt1p to the transcription start site and rendered the *AXL*2 insensitive to nuclear ribonucleases. These observations reveal a model of cell cycle-dependent regulation of gene expression whereby the promoter regulates RNA stability when transcription is not repressed.

Previous studies indicated that promoter sequence may control RNA stability in the cytoplasm^14,39^. In all these examples, accelerated RNA decay is coupled to transcription repression and the degradation events occur in the cytoplasm after transcription is completed. Therefore, in these examples accelerated RNA decay functions to accentuate transcriptional repression. In the case of *AXL2*, the role of RNA decay takes center stage as the promoter is directly responsible for the recruitment and/or activation of the RNA decay machinery to the transcription site (Fig. 7). Most importantly, there was a decrease but no arrest in *AXL2* transcription as Axl2 mRNA decreases (Fig. 1 and Supplementary Fig. 3). Therefore, unlike the Dbf2p and Rap1p promoter-induced decay, the repression of *AXL2* expression is achieved in the nucleus while the gene continues to be transcribed.

It is unlikely that this direct feedback between synthesis and degradation is restricted to *AXL2*. Rnt1p RNase has hundreds of potential cleavage targets in the yeast transcriptome, including in cell cycle genes^20^. Equally, there are at least two other mRNAs coding for protein required for cell polarity and bud site selection (*BEM2* and *BNI1*) that were identified using high-throughput assay as targets for nuclear RNA degradation^20^. However, it remains to be seen if these degradation events act as the primary mechanism of gene regulation or function as fail-safe mechanisms to ensure clean transcription repression. Distinguishing between transcriptional repression and cotranscriptional RNA degradation of transcribed genes is challenging since both mechanisms reduce the amount of RNA and, as we show also, may both depend on the promoter sequence. In addition, bulk measurements of total RNA decay cannot easily differentiate between these two nuclear mechanisms since most mRNA is cytoplasmic. Live cell analysis coupled with single molecule counting and RNAPII ChIP assay, as we performed here, will be required to clearly establish the regulatory mechanism of these candidate genes. Comparing both untagged RNA FISH assay and live cells assay of tagged RNA increases confidence in the data obtained and reduces the chance of technique specific artefacts. In addition, comparing between conditions or ribonuclease deletion using the same probe set or tag reduces the chance of errors of these techniques. In any case, the findings in this study caution against the automatic interpretation of the dependency of gene expression on promoter sequence as evidence for transcription regulation.

RNA stability is regulated by a large network of ribonucleases and blocking the mutation by one may activate the degradation by another. This explains why mutation of the RNA site that Rnt1p cleaves in the presence of exoribonucleases does not increase RNA expression (Supplementary Fig. 7b). RNA accumulating in the nucleus upon the mutation of Rnt1p loop are targets of the DRN or the nuclear RNA degradation mechanism that include a component of the nuclear cap binding complex (Cbc1p) and nuclear ribonucleases like Rrp6p and Rat1p^40,41^. Indeed, deletion of exoribonucleases including Rrp6p increases RNA abundance in a Rnt1p cleavage site-dependent manner (Supplementary Fig. 7b).

**Fig. 8:**
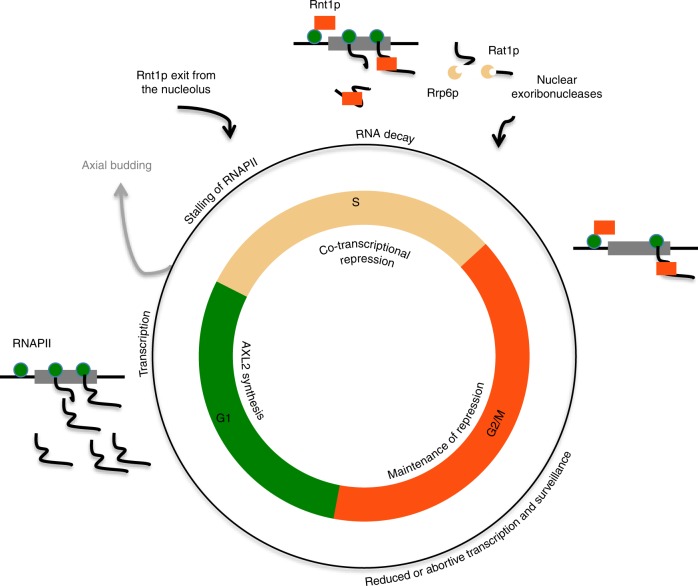
Model for cell cycle-dependent repression of the budding gene *AXL2*. In this model, *AXL2* transcription is induced in the G1 phase of the cell cycle and repressed in S phase when Rnt1p is released from the nucleolus. Association of Rnt1p to the promoter stalls transcription and initiates RNA degradation. Exoribonucleases degrade the RNA cleaved by Rnt1p and function as a fail-safe to remove the RNA escaping cleavage by Rnt1p. In the G2/M phase *AXL2* repression is maintained by a combination of reduced transcription initiation and increased RNA degradation. After mitosis, Rnt1p is re-localized to the nucleolus releasing the RNAPII for rapid transcription of *AXL2*

The data presented in this study suggest a model of cell cycle-dependent gene expression (Fig. 8). In this model, in the absence of Rnt1p, which is mostly nucleolar in the G1 phase of the cell cycle^29^, Axl2 mRNA accumulates and is translated in the cytoplasm. Upon the exit of Rnt1p from the nucleolus to the nucleoplasm in the S phase of the cell cycle, it is recruited to the promoter region of *AXL2* to survey RNA synthesis and cleaves it upon the transcription of the cleavage signal. This cleavage leads to a sharp decrease in the number of complete RNA exported to the cytoplasm and releases RNAPII. It is not clear how Rnt1p is recruited to the promoter. We have shown that the interaction between Rnt1p and the promoter does not depend on the RNA sequence of the open reading-frame. Indeed, disrupting the RNA site that Rnt1p cleaves increases the interaction with the promoter region (Fig. 6). This reinforces a model whereby Rnt1p binds to the promoter region before translocating to the *AXL2* stem loop when it is synthesized. Rnt1p may be recruited by cell cycle-specific transcription factors or directly by interaction with RNAPII itself. Protein interaction assay identified binding of Rnt1p with several transcription factors and interaction with RNAPII CTD was also proposed^30,42^. Unlike standard, transcription-based models of cell cycle-dependent regulation, this RNA decay-dependent mechanism provides faster response time permitting rapid cycling of gene expression.

## Methods

### Yeast strains

Yeast strains were generated in the BY background^43^ starting from strain LLY36^44^ using standard procedures^45^. 24xMS2 tags^23^ were introduced in the 5′ UTR of *AXL2* by transforming a PCR fragment amplified from plasmid pMS2-*AXL2* synthesized by BioBasic. Likewise, 24xPP7 tags^23^ were introduced in the 3′ UTR of *AXL2* by transforming a PCR fragment amplified from plasmid p*AXL2-*PP7, which was synthesized by BioBasic. Strains expressing RNA tags were crossed to BY4720^43^ to remove *TRP1* to use plasmid pNoel-NLSred or pNoel. The LoopR mutant was produced by replacing the cleavage site of Rnt1p in *AXL2* with a GFP cassette. The LoopM mutant was produced by introducing five silent point mutations in the sequence of the cleavage site of Rnt1p in *AXL2* to disrupt its predicted secondary structure without changing the coding sequence. The *AXL2* promoter replacement by the promoter of *TOA1* was performed using a synthesized plasmid as template to amplify a replacement cassette.

### Yeast cultures

All yeast strains were grown in yeast complete (YC) media lacking the appropriate nucleotide or amino acid when needed for plasmid maintenance (1.7 g/L yeast nitrogen base without amino acids and ammonium sulfate, 1 g/L sodium glutamate, 100 mg/L cysteine, 100 mg/L threonine, 85 mg/L tryptophan, 80 mg/L leucine, 60 mg/L lysine, 50 mg/L aspartic acid, 50 mg/L isoleucine, 50 mg/L methionine, 50 mg/L phenylalanine, 50 mg/L proline, 50 mg/L serine, 50 mg/L tyrosine, 50 mg/L uracil, 50 mg/L valine, 20 mg/L adenine, 20 mg/L arginine, 20 mg/L histidine, pH 6) with 2 g/L dextrose. Cultures were grown at 26 °C to accommodate thermosensitive strains such as *rnt1∆* and *rat1-1* unless restrictive conditions were required in which case precultures grown at the permissive temperature were incubated at the restrictive temperature of 37 °C for 4 h prior to cell collection. Cell cycle arrest was performed with cultures grown in YEPD (10 g/L yeast extract, 20 g/L bacto-peptone, 100 mg/L adenine, 2 g/L dextrose) at 30 °C. G1 arrest was performed by adding 5 µg/ml α-Factor to the culture and monitoring the arrest by looking at the budding index of sonicated culture samples. After 3 h of treatment, >95% were un-budded and cells were collected. S phase arrest was performed by the addition of 200 mM hydroxyurea powder to the cultures and monitoring the arrest as for G1. After 3 h of treatment, >90% were budded and cells were collected.

The G2/M arrest was performed by the addition of 15 µg/ml nocodazole from 1.5 mg/ml stock in DMSO to the cultures and monitoring the arrest as above. After 3 h of treatment, >90% of the cells were arrested with large buds and the cells were collected. Transcription arrests were performed in cells grown in YC media at 26 °C. The cultures were shifted to 37 °C for 20 min prior to the addition of 150 µM bathocuprioinedisulphonic acid followed 10 min later by the addition of 10 µg/ml thiolutin with 150 nM CuSO_4_. Samples were collected 0, 5, 10, 20, 40 min after the addition of thiolutin.

### Single-molecule fluorescence in situ hybridization

The yeast smFISH protocol was performed essential as previously described^21^. 50 ml cultures were grown to an OD_600_ of 0.4–0.6 and fixed for 45 min at room temperature by adding 32% electron microscopy-grade formaldehyde to a final concentration of 4%. Fixed cells were washed with buffer B (1.2 M sorbitol, 100 mM KHPO_4_ pH 7.5), digested for 10–20 min in buffer B with 20 mM vanadyl ribonucleoside complex, 20 mM beta-mercaptoethanol and 125–375 U of lyticase adjusted for each strain to achieve >90% cell digestion in that time frame. Digested cells were washed with buffer B and stored at −20 °C in 70% EtOH. 1.5e7 cells were rehydrated in 2× SSC and prehybridized in 2× SSC 10% formamide for 1 h at 37 °C. Hybridizations were carried out in tubes overnight at 37 °C in a final volume of 100 µl containing 2× SSC, 10% formamide, 10% dextran sulfate, and 10 ng of each set of probes. Probe sets consisted either of 12 × 20 nucleotide-long DNA oligos complementary to the 5′ sequence of Axl2 mRNA labeled with DyLight 650, 12 × 20 nucleotide-long DNA oligos complementary to the 5′ sequence of Axl2 mRNA labeled with DyLight 550, or 4 × 20 nucleotide-long DNA oligos complementary to the internal spacer “2” sequence of the pre-ribosomal RNA labeled with Alexa 488 used as a hybridization control. Probes sequences were chosen using the Stellaris Probe Designer web tool and synthesized by Biosearch technologies. After hybridization, cells were washed once with 2× SSC 10% formamide, once with 2× SSC 0.1% Triton, and once with PBS. 2E5 stained cells were deposited on poly-lysine coated coverslips, allowed to settle for 30 min, and washed with EtOH prior to mounting on a slide with ProLong Gold with DAPI. Slides were sealed after an overnight curing at room temperature. Visualization was performed on a Zeiss microscope with an oil immersion alpha Plan-Apochromat 100×/1.46 objective. A Zeiss mRM camera was used and *z*-stacks of 20 images spaced by 200 nm were collected with Zen 2012 software. DAPI was illuminated for 200 ms with a 365 nm LED and viewed through a filter cube 62HE, DyLight 550 was illuminated for 2 s with an HXP lamp and viewed through a filter cube 43HE. DyLight 650 was illuminated for 4 s with a 625 nm LED and viewed through filter cube 77HE. For display purposes, the image stacks were deconvoluted using the Zen 2012 constrained iterative algorithm and the maximum projection was presented in the pictures.

### Live cell imaging

Yeast cultures expressing MS2 repeats in the 5′ UTR of Axl2 mRNA and containing a plasmid encoding MCP-GFP and NLS-td-tomato were grown to an OD_600_ of 0.4–0.6, concentrated by centrifugation to an OD_600_ of 2 and spotted on 1.2% agarose pads made with growth media. Cells expressing MS2-*AXL2* or *AXL2*-PP7 and with a plasmid containing MCP-GFP and PCP-td-tomato were grown and prepared likewise. The coverslips were sealed with petroleum. Slides were visualized in a 26 °C incubation chamber on a Zeiss spinning disk microscope with an oil immersion alpha Plan-Apochromat 100×/1.46 objective. An Evolve 512 camera with EM gain set at 500 was used and *z*-stacks of 5–7 images spaced by 500 nm were collected using the Zen 2012 software. GFP was illuminated for 100 ms with a 100 mW 488 nm laser set at 10–20% through a 500–550 nm emission filter. Td-tomato was illuminated for 100 ms with a 40 mW 561 nm laser set at 20% through a 598–660 nm emission filter. The stacks of images were converted to 2D images by maximum projection and exported with the Zen 2012 software. RNA molecules were counted manually using the profile analysis tool in Zen 2012 on maximum projected images and scored when spots were twice as bright as the cellular background. Brightness and contrast were adjusted using Photoshop CS6 to set a common background level to the time series, for display purposes. The arbitrary cut-off was determined by contrast adjustment of live cell images to identify spots comparable in size to those observed by FISH. To compensate for the higher background of the live cell images in the GFP channel compared to the DyLight 550 and DyLight 650 channels of FISH we only counted GFP spots with SNR >2.

### Budding scar analysis

2E6 cells fixed as for FISH were washed once with water and resuspended in 1 mg/ml Calcofluor and stained for 5 min at room temperature. Stained cells were washed twice with water and 4E5 cells were deposited on ConA coated coverslips, allowed to settle for 30 min, rinsed with water and mounted wet. Visualization was performed under a Zeiss microscope with an alpha Plan-Apochromat 100×/1.46 oil-immersion objective. A Zeiss mRM camera was used and *z*-stacks of 20 images spaced by 200 nm were collected with Zen 2012 software. Calcofluor was illuminated for 100 ms with a 365 nm LED and viewed through filter cube 62HE. The images were deconvoluted with the Zen 2012 constrained iterative algorithm and the maximum projection was presented in the figures.

### RNA isolation

Yeast cultures were grown to an OD_600_ of 0.4–0.6 and 50 ml of culture was collected by centrifugation, washed with ice-cold water and resuspended in 300 µl LETS buffer (100 mM LiCl, 10 mM EDTA, 10 mM Tris, 0.2% SDS, pH 7.5) with 350 µl phenol and 750 µl acid-washed glass beads. Cells were broken by 7 cycles of vortexing for 30 s followed by 30 s on ice. The lysates were extracted 2 times with phenol: chloroform: isoamyl alcohol (25:24:1) and once with chloroform: isoamyl alcohol (24:1) before ethanol precipitation.

### In vitro cleavage of total RNA by Rnt1p

Cleavage reactions were performed on 25 µg of total RNA extracted from *rnt1*⊗ cells in a total volume of 100 µl for 20 min at 30 °C. The RNA and 6 pmol of purified enzyme were premixed in reaction buffer (30 mM Tris pH 7.5, 150 mM KCl, 5 mM spermidine, 0.1 mM DTT, 0.1 mM EDTA) and the reactions were started by adding MgCl_2_ to a final concentration of 10 mM. Reactions were stopped by the addition of 1 volume of LETS buffer (described in the “RNA isolation” section) followed by phenol: chloroform: isoamyl alcohol (25:24:1) extraction.

### Northern blot analysis

RNA samples of 20 µg were separated on 1% agarose gels containing 120 mM formaldehyde in MOPS buffer and transferred by capillarity to a charged nylon membrane. Transferred RNAs were bound to the membrane by UV-crosslinking and revealed by methylene blue staining. PCR fragments were used as templates to synthesize ^32^P randomly labeled DNA probes that were hybridized to the membranes overnight at 55 °C in 600 mM NaCl, 120 mM Tris, 8 mM EDTA, 0.2% Ficoll 400, 0.2% polyvinylpyrrolidone, 0.2% BSA, 0.1% SDS, 0.25 mg/ml fragmented herring sperm DNA, pH 8. Membranes were washed twice at 55 °C twice in 600 mM NaCl, 120 mM Tris, 8 mM EDTA, 0.1% SDS, pH 8 and once with 450 mM NaCl, 90 mM Tris, 6 mM EDTA, 0.1% SDS, pH 8, then exposed for 48 h to phosphoimaging screens and revealed on a GE Storm 860 scanner.

### RT-qPCR

Contaminant genomic DNA was removed by DNase treatment of 25 µg of total RNA using 33 units of RNase-free DNase for 25 min at 37 °C on RNeasy spin columns. The treated RNA was eluted from the columns and 50 ng was used as template for reverse transcription using RT Transcriptor from Roche and random hexamers. The cDNA was diluted 15-fold in water and 3 µl of the dilutions were used as template for the qPCR reactions. The qPCR reactions were performed in 10 µl with BioRad iTaq Universal SYBR Green Supermix and 200 nM of primers in 96-well plates on an Eppendorf realplex^2^ Mastercycler epgradient S.

### Chromatin immunoprecipitation

100 ml cultures were grown to OD_600_ 0.4–0.6 and fixed for 20 min at room temperature by the addition of 1% formaldehyde to the culture. The crosslinking reaction was stopped by the addition of 3.6 mM unbuffered Tris and 360 mM glycine. The cell pellets were washed in ice-cold TBS (20 mM Tris, 150 mM NaCl, pH 7.5), split into two aliquots and kept frozen at −80 °C. Frozen pellets were resuspended in 500 µl lysis buffer (50 mM HEPES-KOH pH 7.5, 150 mM NaCl, 1 mM EDTA, 1% Triton X-100, 0.1% Na-deoxycholate) with proteases inhibitors (1 mM PMSF, 1 mM benzamidine, 1 µg/ml Aprotinin, 1 µg/ml Leupeptin, 1 µg/ml Pepstatin A, 1 µg/ml Antipain) and 500 µl acid-washed glass beads, and lysed using a Bertin Precellys 24 with 6 rounds of 10 s of shaking at 6500 rpm, spaced by 60 s pauses on ice. The lysates were adjusted to 750 µl with lysis buffer and sonicated 12 times for 10 s at 20% power amplitude with a Branson digital sonifier spaced by 2 min pauses on ice. The sonicated lysates were cleared by centrifugation at 13,000*g* for 10 min at 4 °C and 500 µl of cleared lysates were mixed with magnetic beads coated with the appropriate primary antibody and incubated overnight at 4 °C with rotation. For RNAPII ChIP, 50 µl of Pan mouse IgG Dynabeads (ThermoFisher Scientific, #11041) were coupled to 3 µg of purified mouse anti-RNA Polymerase II RPB1 antibody (BioLegend, #664906, clone 8WG16). For Rnt1p ChIP, 30 µl of M-280 sheep anti-rabbit IgG Dynabeads (ThermoFisher Scientific, #11203D) were coupled to 3 µl of pre-cleared anti-Rnt1p antibody. The next day, the beads were washed twice with lysis buffer, twice with high salt buffer (50 mM HEPES-KOH pH 7.5, 500 mM NaCl, 1 mM EDTA, 1% Triton X-100, 0.1% Na-deoxycholate), twice with wash buffer (10 mM Tris pH 8, 250 mM LiCl, 1 mM EDTA, 0.5% Igepal CA-630, 0.5% Na-deoxycholate) and once with TE (10 mM Tris pH 8, 1 mM EDTA). The de-crosslinking and elution of DNA was performed by re-suspending the beads in 100 µl TE containing 1% SDS and incubating overnight at 65 °C. Input DNA was de-crosslinked at the same time by incubation of samples of the cleared lysate diluted in TE containing 1% SDS overnight at 65 °C. The DNA was purified by RNase A and proteinase K treatments followed by 2 phenol:chloroform:isoamyl alcohol (25:24:1) extractions, chloroform:isoamyl alcohol (24:1) extraction, and ethanol precipitation with glycogen. The purified DNA pellets were resuspended in 10 mM Tris pH 8 and further diluted in water at 1:200 for the input DNA, 1:20 for the RNAPII ChIP, 1:10 for the Rnt1p ChIP.

### Quantification and statistical analysis

*smFISH analysis*: smFISH experiments were performed on 3 independent cultures and a target of 50 cells per phase were scored coming from each culture. In the figures, all data points are presented in the form of  violin plots unless stated otherwise in the legends and Supplementary Data 1. In Supplementary Fig. 3, the frequency plots show the mean from the 3 cultures (each 50 cells). FISH analysis was performed on the maximum projection of the raw images adjusting for background as described below. 2D images (maximum projections) were imported on a Perkin Elmer Columbus server and analyzed as follows. The nuclei were detected using the DAPI staining and cytoplasms were identified from the cellular background using the orange filter. Cells touching the borders of the image as well as cells smaller than 2 µm^2^ or larger than 20 µm^2^ were removed, while cells with a nucleolar pre-rRNA control signal were kept. Spots were detected and quantified for the 5′ and 3′ probes, then their intensity (corrected to local background) and localization data was exported in a table format. After analysis of the 2D images, cells were manually assigned to G1, S, or G2/M phases of the cell cycle according to the bud size and nucleus position. The value of fluorescence intensity corresponding to 1 RNA molecule was established for each strain by taking the median value of the cytoplasmic RNAs of for all its dataset. The RNA molecule number per spot was calculated by dividing the spot fluorescence intensity by the value corresponding to 1 RNA molecule and rounding to the closest integer. As the primary object detection was based on nuclei identification, many G2/M cells had to be reconstructed from two cells identified from dividing nuclei. Nascent RNA per cell numbers were calculated from the brightest 5′ probes spot in the nucleus when more than 1 nuclear spot was detected. Cytoplasmic RNAs were scored when a spot detected with the 5′ probe also scored positive for the 3′ probe signal.

*RT-qPCR and chromatin immunoprecipitation analysis*: Relative mRNA quantification was achieved through normalization to the Act1 mRNA using the formula: RQ = 2(CT_WT_−CT_mutant_)_Axl2_/2(CT_WT_−CT_mutant_)_Act1_, where CTs are the cycle threshold values determined by the Mastercycler software. PCR reactions were performed in technical duplicates or triplicates for cDNA produced from 3 independent cultures for both the wild-type (WT) and mutant strains. Relative enrichment of RNAPII or Rnt1p at the *AXL2* locus was achieved through normalization to a region of the chromosome V using formula: RE = 2(CT_input_−CT_ChIP_)_Probe_/2(CT_input_−CT_ChIP_)_ChrV_. PCR reactions were performed in technical duplicates or triplicates for ChIP performed at least 2 times in independent cultures.

*Statistics and reproducibility*: Statistical analyses of mean values of measurements were conducted using GraphPad Prism version 7.03 for Windows using Student’s *t* test if so stated in the figure legends with Welch’s correction for FISH data comparison, where variance between strains was unequal. The number of cells analyzed per FISH experiment, means and fold changes to wild-type with *p*-values are reported in Supplementary Data 1. Experiments were repeated 3 times unless stated otherwise.

### Reporting summary

Further information on research design is available in the Nature Research Reporting Summary linked to this article.

## Supplementary information

Reporting Summary

Supplementary Information

Description of additional supplementary items

Supplementary Data 1

Supplementary Data 2

Supplementary Data 3

## Data Availability

All gels and microscopy images are available at Mendeley 10.17632/dx8j25djrw.1. Statistical analysis of the smFISH dataset, genotypes of strains, and primer sequences used in this study are presented in Supplementary Data 1. The source data underlying all figures are provided in Supplementary Data 2. The source data collected from smFISH experiments are shown in Supplementary Data 3.
