# Supplementary material for: Promoter-dependent nuclear RNA degradation ensures cell cycle-specific gene expression
Source: Commun Biol. 2019 Jun 17;2:211. doi: 10.1038/s42003-019-0441-3 (PMC6572803; doi:10.1038/s42003-019-0441-3)
Supplement: Supplementary file 1 — Reporting Summary [file 42003_2019_441_MOESM1_ESM.pdf]

nature research

Corresponding author(s): Sherif Abou Elela
April 19, 2019

Reporting Summary

Nature Research wishes to improve the reproducibility of the work that we publish. This form provides structure for consistency and transparency in reporting. For further information on Nature Research policies, see Authors & Referees and the Editorial Policy Checklist.

Statistical parameters

When statistical analyses are reported, confirm that the following items are present in the relevant location (e.g. figure legend, table legend, main text, or Methods section).

n/a	Confirmed	
☐	☒	The exact sample size (*n*) for each experimental group/condition, given as a discrete number and unit of measurement
☐	☒	An indication of whether measurements were taken from distinct samples or whether the same sample was measured repeatedly
☐	☒	The statistical test(s) used AND whether they are one- or two-sided<br>*Only common tests should be described solely by name; describe more complex techniques in the Methods section.*
☒	☐	A description of all covariates tested
☐	☒	A description of any assumptions or corrections, such as tests of normality and adjustment for multiple comparisons
☐	☒	A full description of the statistics including central tendency (e.g. means) or other basic estimates (e.g. regression coefficient) AND variation (e.g. standard deviation) or associated estimates of uncertainty (e.g. confidence intervals)
☐	☒	For null hypothesis testing, the test statistic (e.g. *F*, *t*, *r*) with confidence intervals, effect sizes, degrees of freedom and *P* value noted<br>*Give P values as exact values whenever suitable.*
☒	☐	For Bayesian analysis, information on the choice of priors and Markov chain Monte Carlo settings
☒	☐	For hierarchical and complex designs, identification of the appropriate level for tests and full reporting of outcomes
☒	☐	Estimates of effect sizes (e.g. Cohen's *d*, Pearson's *r*), indicating how they were calculated
☐	☒	Clearly defined error bars<br>*State explicitly what error bars represent (e.g. SD, SE, CI)*

Our web collection on statistics for biologists may be useful.

Software and code

Policy information about availability of computer code

Data collection	Zen 2012 Blue Edition (Zeiss), Realplex version 1.5 (Eppendorf), Storm Scanner Control version 5.03 (Amersham Biosciences)
Data analysis	Zen 2012 Blue Edition (Zeiss), Columbus version 2.5.1 (Perkin Elmer), Photshop CS6 (Adobe), Realplex version 1.5 (Eppendorf) Prism version 7.03 (GraphPad Software)

For manuscripts utilizing custom algorithms or software that are central to the research but not yet described in published literature, software must be made available to editors/reviewers upon request. We strongly encourage code deposition in a community repository (e.g. GitHub). See the Nature Research guidelines for submitting code & software for further information.

Data

Policy information about availability of data

All manuscripts must include a data availability statement. This statement should provide the following information, where applicable:

- Accession codes, unique identifiers, or web links for publicly available datasets
- A list of figures that have associated raw data
- A description of any restrictions on data availability

All gels and raw microscopy images presented in Fig. 1, 2, 3, 4, 5, 6 and 7 as well as supplementary Fig. 1, 2, 4, 6, 7 and 9 are davailable in Mendeley https://data.mendeley.com/datasets/dx8j25djrw/draft?a=c24cdf2e-5915-401c-8a8b-6bea84bd3fbc

Field-specific reporting

Please select the best fit for your research. If you are not sure, read the appropriate sections before making your selection.

☒ Life sciences ☐ Behavioural & social sciences ☐ Ecological, evolutionary & environmental sciences

For a reference copy of the document with all sections, see nature.com/authors/policies/ReportingSummary-flat.pdf

Life sciences study design

All studies must disclose on these points even when the disclosure is negative.

Sample size	For FISH analysis, 50 cells from each phase of the cell cycle were analyzed. The experiment was repeated 3 times with independant cultures. Less cells were used when the mutants or conditions used caused bias in the cell cycle distribution and prevented data collection. Exact n used for every FISH analysis is stated in supplementary table 1.
Data exclusions	For FISH analysis, cell permeability to FISH probes was tested with a pre-rRNA probe that labeled the nucleolus. Cells that were not stained with this control probe were discarded from the analysis.<br>For FISH analysis, cells were manually annotated to cell cycle phases in accordance with their budding state and nuclear position. Cells that were clustered in ways that prevented unambiguous annotation of the cell cycle were excluded from the analysis.
Replication	Data were collected from 3 independent cultures unless stated otherwise.
Randomization	No sample randomization was done. Strains of the same background with specific gene mutations were used. When mutants were expressed on plasmids, the WT plasmid was used as a control. When thermosensitive mutants were used, the permissive and repressive temperature were compared.
Blinding	No blinding was done in order to keep track of the samples and strains used in this study.

Reporting for specific materials, systems and methods

Materials & experimental systems

n/a	Involved in the study
☐	☒ Unique biological materials
☐	☒ Antibodies
☒	☐ Eukaryotic cell lines
☒	☐ Palaeontology
☒	☐ Animals and other organisms
☒	☐ Human research participants

Methods

n/a	Involved in the study
☒	☐ ChIP-seq
☒	☐ Flow cytometry
☒	☐ MRI-based neuroimaging

Unique biological materials

Policy information about availability of materials

Obtaining unique materials	All unique materials are available from the authors.

Antibodies

Antibodies used	Purified anti-RNA Polymerase II RPB1 mouse antibody, BioLegend cat#664906, clone 8WG16, lot# B232736<br>Polyclonal anti-Rnt1p rabbit antibody
Validation	The anti-RNA Polymerase II RPB1 antibody from clone 8WG16 has been used for chIP against S.cerevisiae RPB1 and compared to other RNA Polymerase II antibodies in Bataille et al. 2012 Molecular Cell (pmid 22284676). BioLegend tests the antibody by Western blot.<br>Anti-Rnt1p antibody was produced by injection of afinity purified recombinant Rnt1p in rabbits (Cocalico Biologicals, Reamstown, PA) and was first described in Tremblay et al. 2002 Mol Cell Biol. (pmid 12052886). Serum was preincubated with protein extract from yeast cells not expressing Rnt1p. Validation was done by Western blot against protein extracts from cells expressing Rnt1p or not.
