# Supplementary material for: Promoter-dependent nuclear RNA degradation ensures cell cycle-specific gene expression
Source: Commun Biol. 2019 Jun 17;2:211. doi: 10.1038/s42003-019-0441-3 (PMC6572803; doi:10.1038/s42003-019-0441-3)
Supplement: Supplementary file 2 — Supplementary Information [file 42003_2019_441_MOESM2_ESM.pdf]

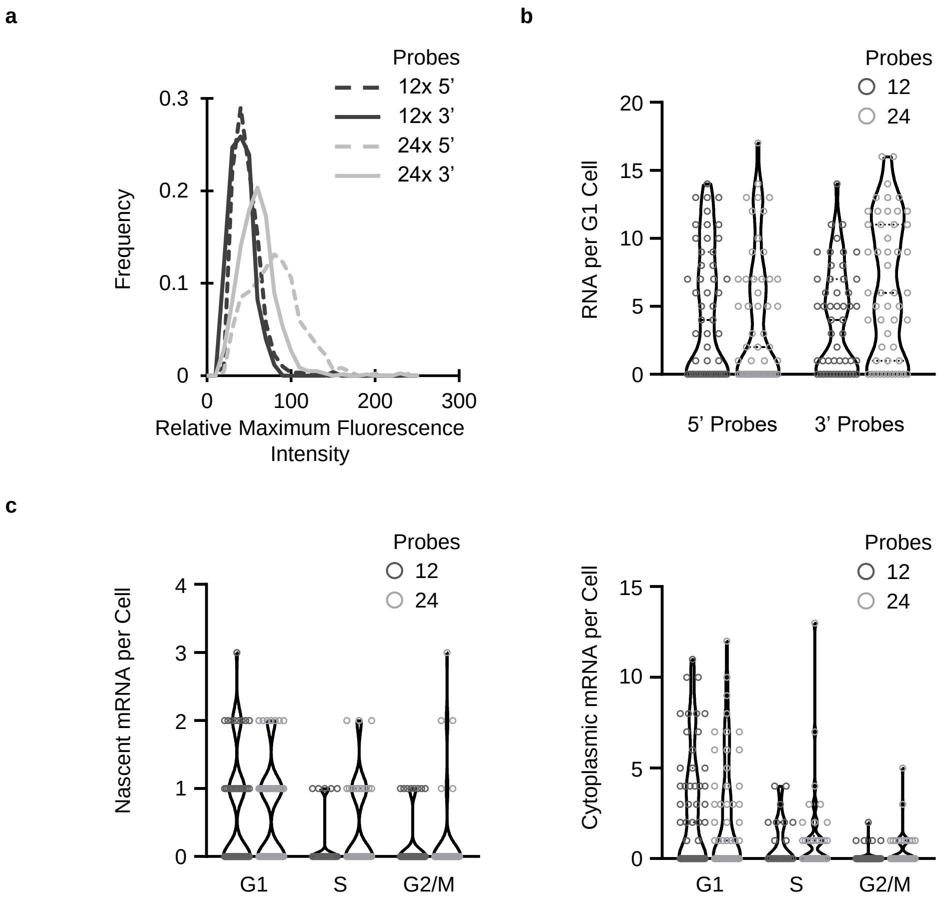

Supplementary Figure 1 (related to Fig. 1b). Effect of FISH Probe Number on Signal Intensity and Number of RNA Detected

a, Comparison of the fluorescence profiles obtained using different numbers of probes. Series of 12 or 24 single labeled oligonucleotides hybridizing to the first 323 (12x 5’ probes) or 848 (24x 5’ probes) nucleotides and to the last 370 (12x 3’ probes) or 1249 (24x 3’ probes) nucleotides of Axl2 mRNA were used in FISH and the maximum signal of each cytoplasmic spot relative to its surrounding was recorded as relative maximum fluorescence intensity.

b, Effect of probes number on the number of RNA detected. The number of RNA per cell was calculated for 50 G1 cells using the medians of the cytoplasmic spots relative maximum fluorescence intensity (39 for 12x 5’ probes, 75 for 24x 5’ probes, 35 for 12x 3’ probes and 54 for 24x 3’ probes) as a reference for the value of 1 RNA.

c, Comparison of Axl2 mRNA expression pattern obtained using different number of probes. Nascent transcripts per cell (left panel) were calculated from the brightest spot found in the nucleus when more than one nuclear spot were found. Cytoplasmic mRNAs (right panel) were calculated from spots with a signal ≥ 1 RNA for both 5’ (12 or 24) and 3’ (12) probes. Data from 50 cells was used for G1 and between 20 and 50 cells for S and G2/M data.

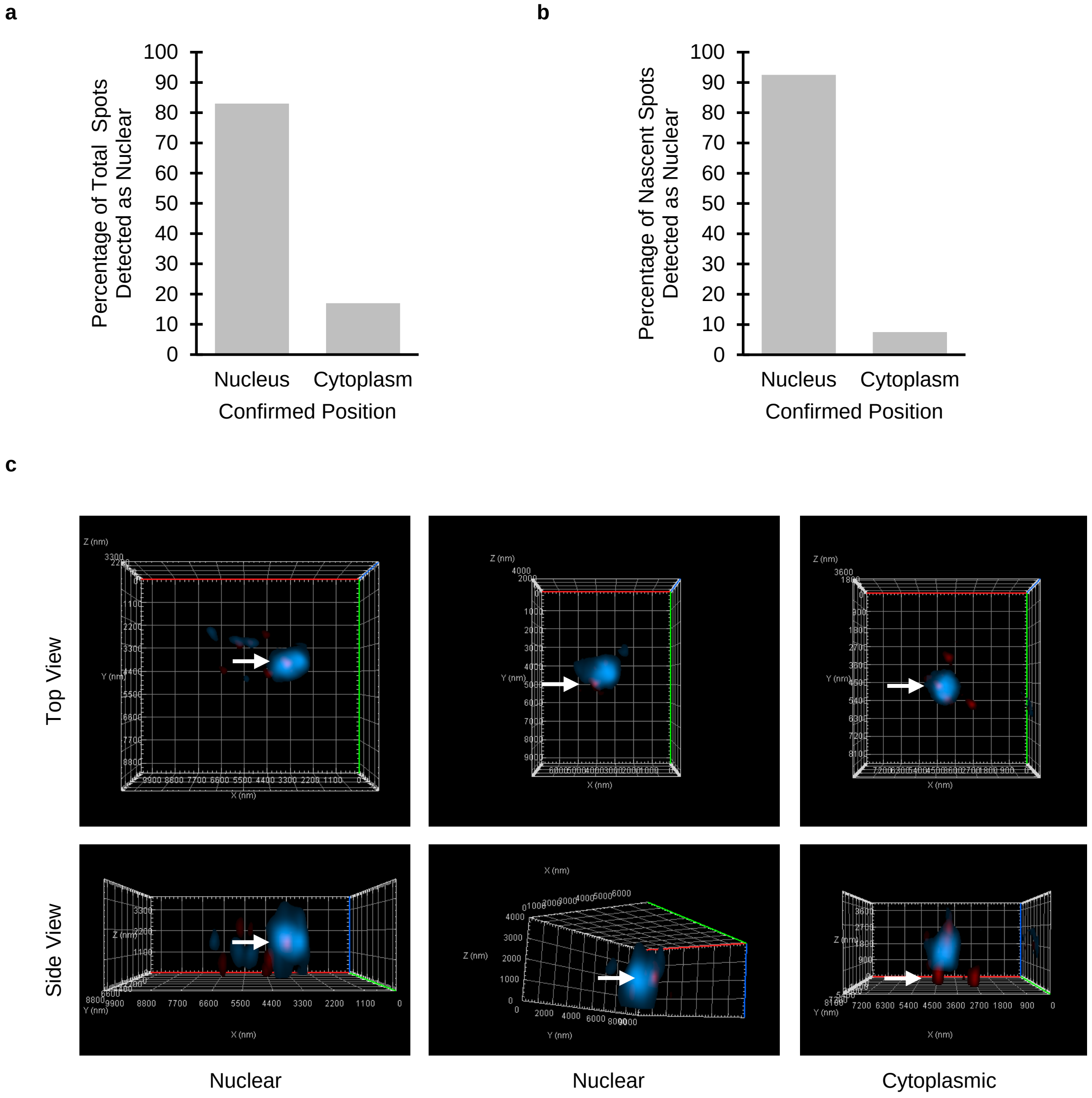

Supplementary Figure 2 (related to Fig. 1b). Effect of Performing the RNA Quantification on 2D Projections of 3D Image Data

a, Manual confirmation of the position of nuclear RNA sports using 3D data. The position of the nuclear transcripts detected in stacked images was confirmed manually in the original 3D data to evaluate the false positives due to cytoplasmic RNAs found above or below the nucleus. The manual assessment was done for 40 wild type cells with 53 detected nuclear spots.

b, Manual confirmation of the position of transcription spot using 3D data. When more than one spot is detected in the nucleus the brightest spot was considered the transcription spot and its nuclear location was confirmed manually using uncompressed images as described in the Methods section.

c, Examples of the top and side view of spots detected as nuclear in compressed 2D images. The real nuclear and cytoplasmic location of the different spots is indicated at bottom. As shown two of the examples on the left show nuclear spots and one example on the right show a cytoplasmic spot that was mislabelled by the 2D analysis as nuclear. The white arrows point to the spots of interest. Scales in nm are represented on the images.

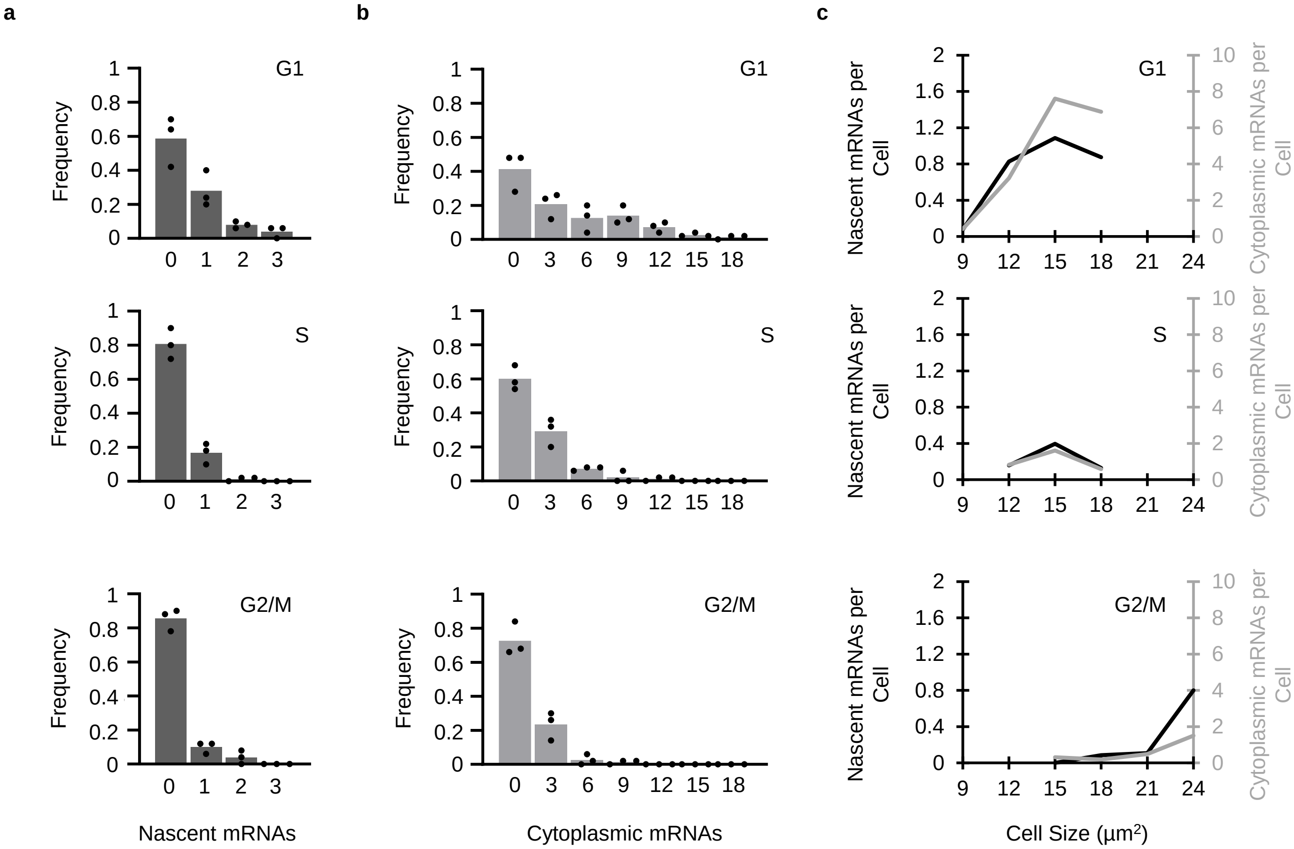

Supplementary Figure 3 (related to Fig. 1b). Axl2 mRNA Synthesis Continues After the G1 Phase of the Cell Cycle

a, Distribution of the number of Axl2 mRNA detected in the nucleus in different phases of the cell cycle. Quantification of nascent Axl2 mRNA in the G1, S and G2/M phases of the cell cycle. Nascent transcripts were counted using 12 probes hybridizing to the mRNA 5' end at the transcription site. The frequency by which 1, 2 or 3 transcripts were detected per cell was calculated for 50 cells in and the mean frequency for 3 biological replicas was plotted in the form of a bar graph.

b, Quantification of the number of cytoplasmic Axl2 mRNA in each phase of the cell cycle. Complete mRNA (i.e. detected by colocalized 5' and 3' signals) in the cytoplasm was quantified as described as in (**a**).

c, Cell size dependent distribution of Axl2 mRNA. The number of nascent and cytoplasmic mRNAs in the different phases of the cell cycle was calculated as in (**a** and **b**) and plotted according to the cell size separated in bins of 3 μm^2. Data points representing less than 5% of the data (< 8/150 cells) were not shown.

a

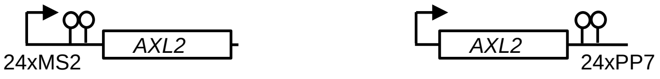

b

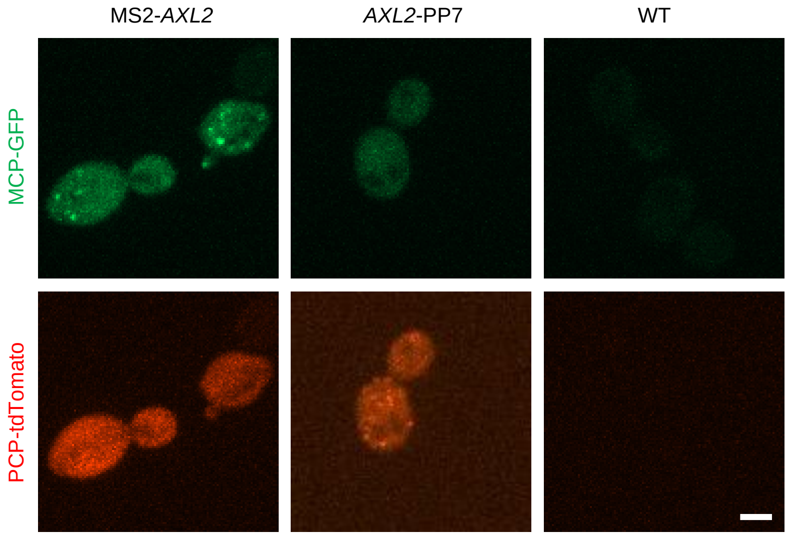

Supplementary Figure 4 (related to Fig. 2). MS2-Axl2 Detection is Dependent on the Presence of the MS2 Repeats

a, Schematic representation of *AXL2* tagging strategy. Axl2 mRNA was tagged with either MS2 or PP7 coat protein binding sites. The 24x MS2 repeats were inserted in the 5 'UTR sequence of *AXL2* (MS2-*AXL2*) or the 24x PP7 (*AXL2*-PP7) repeats were inserted in the 3' UTR sequence of *AXL2*.

b, Examples of cells expressing MS2 or PP7 tagged Axl2 mRNA. Strains transformed with plasmid pNoel expressing either MCP-GFP and PCP-tdTomato were visualized using GFP (shown in green) and tdTomato (shown in red). Green spots were only detected with GFP only when MS2 tag is present (top left panel), while tdTomato detected spots (shown in red) only when the PP7 tag was present (bottom middle panel). Example of the background generated in the absence of the tag (top middle and bottom left panels) or the absence of GFP and tdTomato (right panels) are shown. White bar = 2μm.

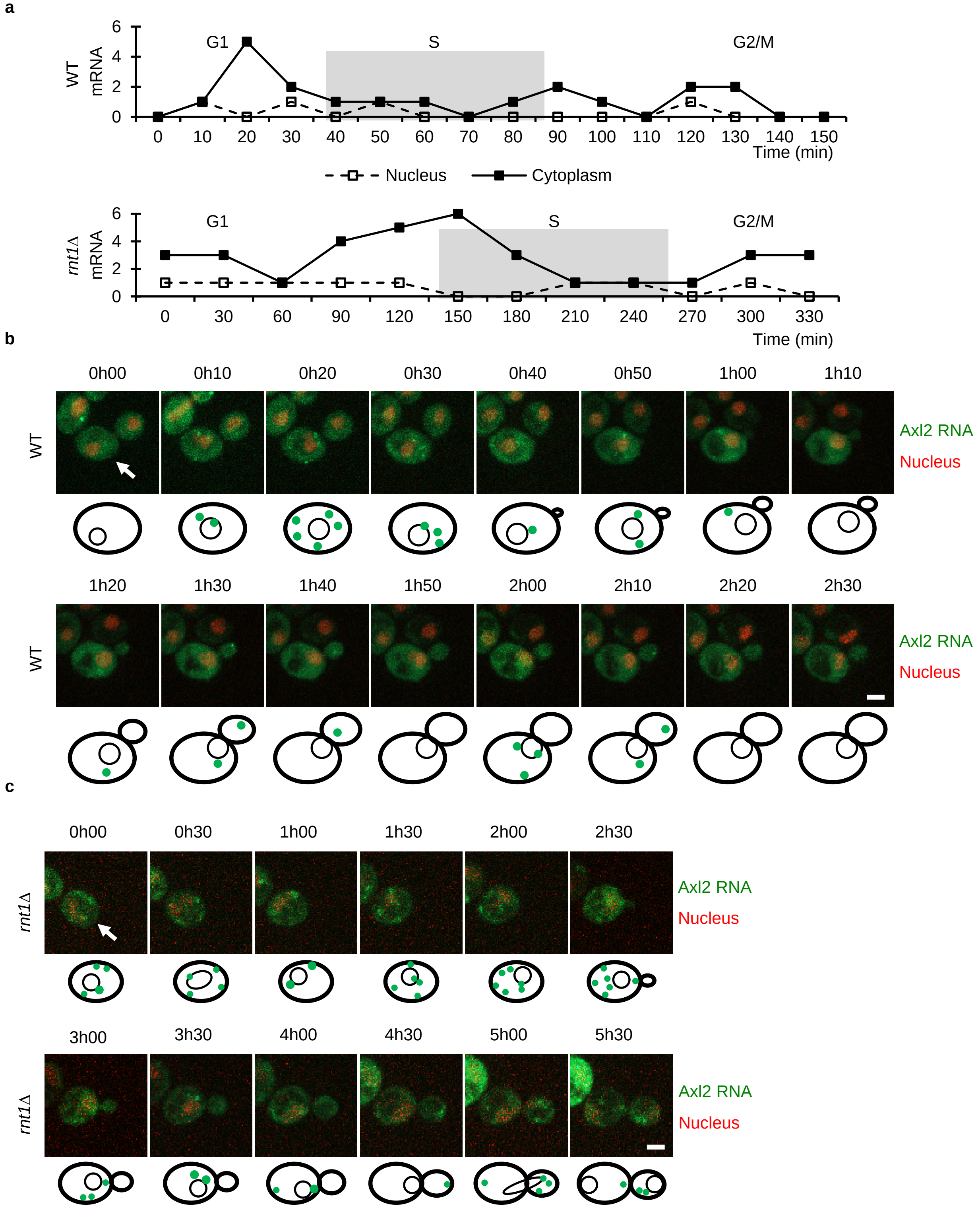

Supplementary Figure 5 (related Fig. 2). Rnt1p Cleavage Triggers Rapid Repression of *AXL2* Expression in the S Phase of the Cell Cycle

a, Comparison of the detailed timeline of MS2-tagged Axl2 mRNA expression in live WT and *rnt1*Δ cells. Axl2 was detected in different time in live cells passing through different phases of the cell cycle as described in Figure 5a.

b, Example of the images obtained for live WT cells with the nucleus in red and MCP-GFP to reveal MS2-tagged Axl2 mRNA were imaged every 10 minutes. White bar = 2μm. Time frames are shown with cartoon tracings of the cell of interest below.

c, Example of the images obtained for live *rnt1*Δ cells. The images are presented as in (**b**) with time frames acquired every 30 minutes.

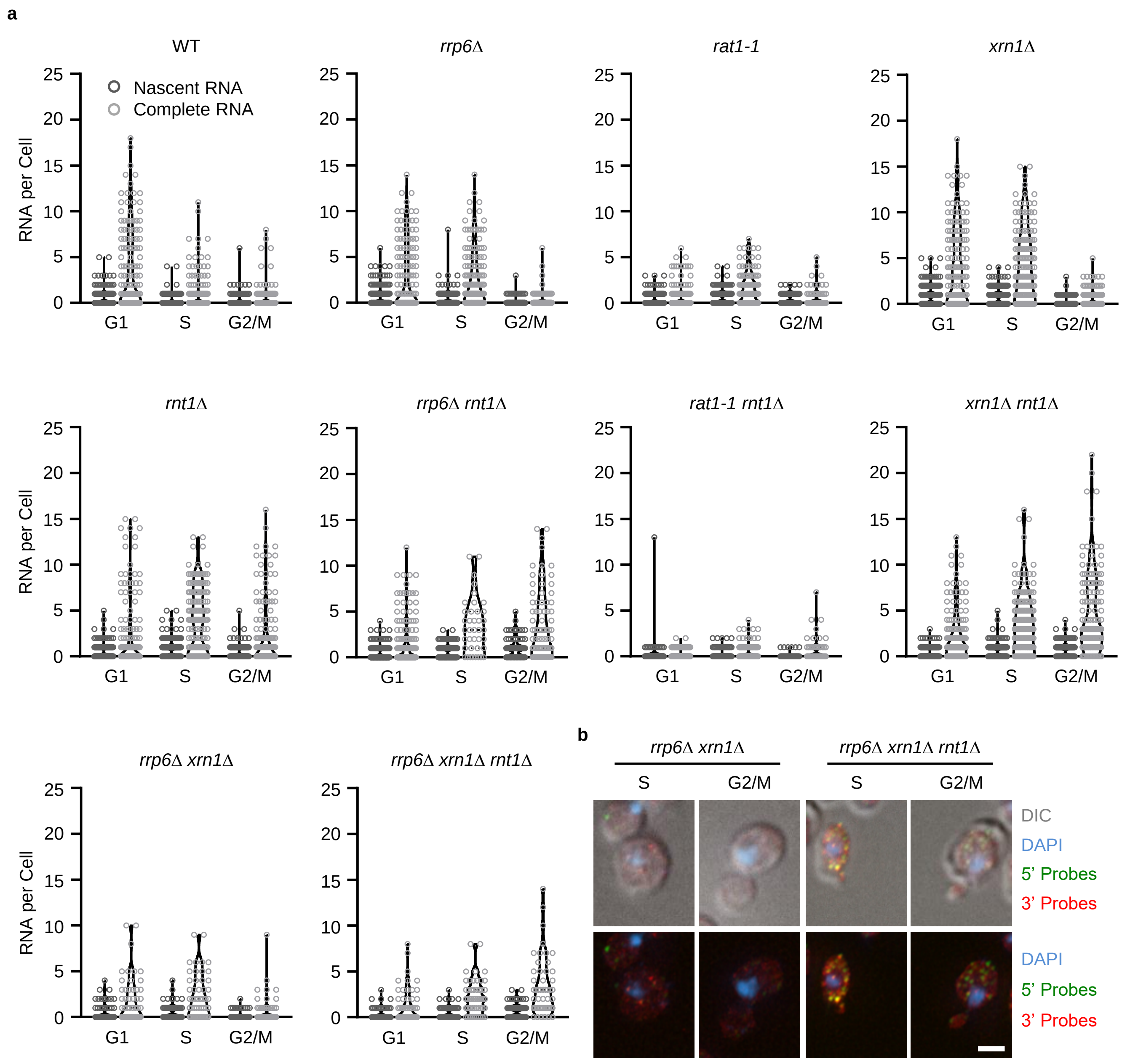

Supplementary Figure 6 (related to Fig. 4). Nuclear Endonucleases Induce Cell Cycle Dependent Repression of *AXL2* Expression

a, Impact of single, double and triple ribonuclease deletion on the cell cycle dependent expression of Axl2 mRNA. The number of nascent (dark grey) and complete cytoplasmic (light grey) transcripts was calculated in different phases of the cell cycle for each ribonuclease deletion strain using smFISH as described in Figure 1C. The strain names are indicated on top. The wild type strain is included as reference for comparison. The count for nascent RNA (RNA detected only with the 5' end probes in the nucleus) is shown in dark gray and the count of cytoplasmic RNA (RNA detected by both 5' and 3' end probes in the cytoplasm) is shown in light gray.

b, Multiple ribonuclease deletions strongly inhibit the repression of *AXL2* in the G2/M phases of the cell cycle. Examples of the images showing the impact of deleting two and three ribonucleases on the accumulation of Axl2 mRNA in the S and G2/M phases of the cell cycle. Axl2 mRNA was detected in cells lacking *RRP6* and *XRN1* or *RRP6*, *XRN1* and *RNT1* ribonucleases in the S and G2/M phases of the cell cycle. The probes and stains used are shown on the right. RNA hybridizing to the 5' end probes only are shown in green, those hybridizing to the 3' end probes only in red and those hybridizing to both in yellow. The merged probe images are shown at bottom and those merged with DIC on top. The white bar equals 2 μm.

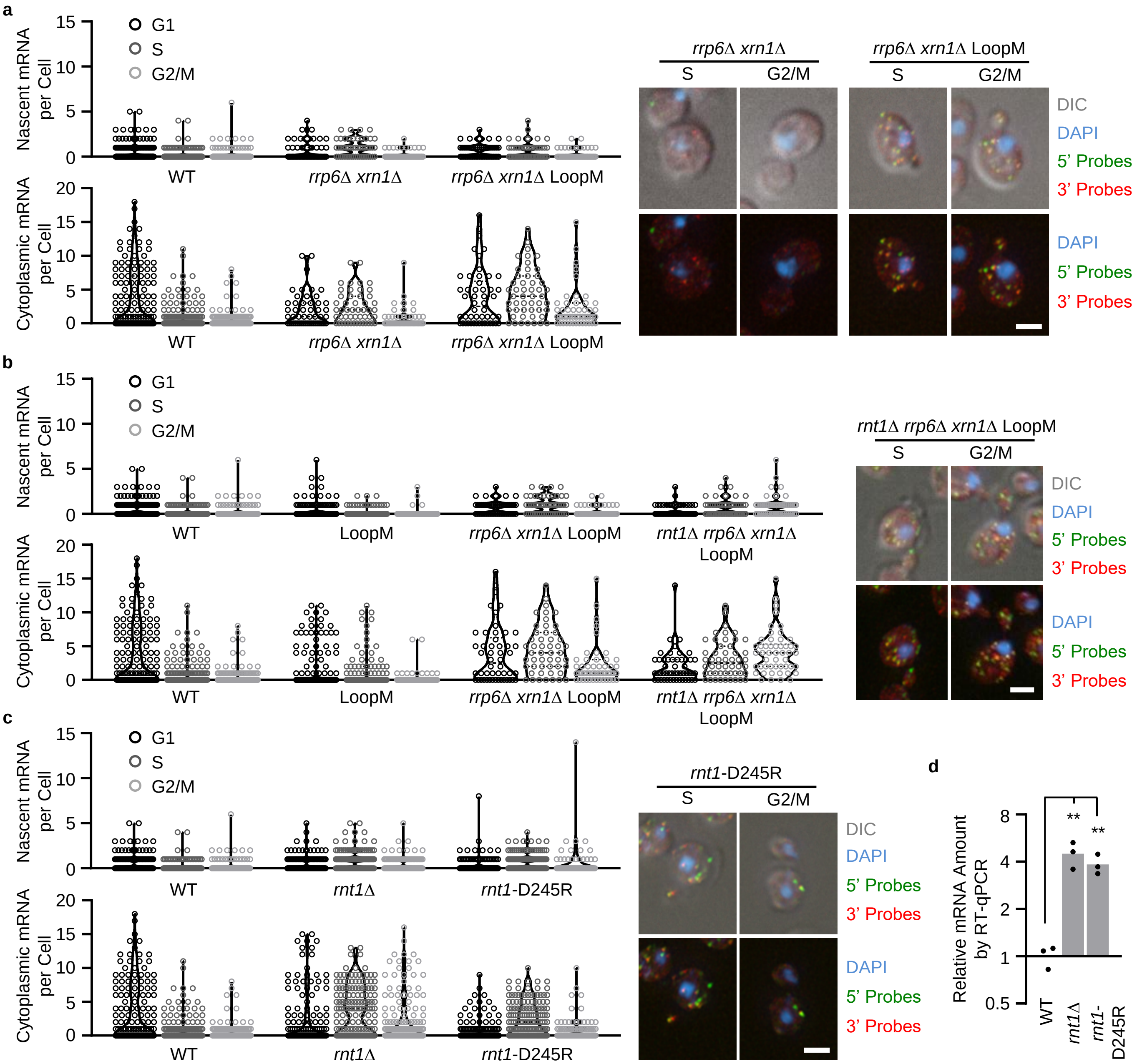

Supplementary Figure 7 (related to Fig. 4). Rnt1p Regulates *AXL2* Expression in Both Cleavage Independent and Dependent Manner

a, Mutations in Rnt1p cleavage site impair the degradation of Axl2 mRNA in the absence of *RRP6* and *XRN1*. Axl2 mRNA expression pattern was compared in cells lacking the *RRP6* and *XRN1* and cells carrying both deletions and silent mutations in the cleavage site of Axl2 (LoopM). The amount of nascent (top) and cytoplasmic RNA (bottom) were quantified and shown in the form of violin plots. Examples of S and G2/M cells are shown in the right panels.

b, Deletion of *RNT1* do not affect the expression of RNA with mutated cleavage site except in the G2/M phase of the cell cycle. *RNT1* was deleted in the presence and the absence of loop mutations in different ribonucleases background to distinguish between cleavage dependent and independent effects. Axl2 mRNA was quantified by FISH in WT, LoopM, *rrp6Δ xrn1Δ* LoopM and *rnt1Δ rrp6Δ xrn1Δ* LoopM cells. Examples of *rnt1Δ rrp6Δ xrn1Δ* LoopM cells in S and G2/M phases of the cell cycle are presented in the right panel. White bars equal 2 µm.

c, The catalytic activity of Rnt1p is required for the repression of *AXL2* in the S phase of the cell cycle. Axl2 mRNA was monitored in wild type cells or cells expressing a mutated version of Rnt1p that can bind but not cleave RNA (*rnt1*-D245R).

d, Inactivation of Rnt1p catalytic activity increases the overall expression level of Axl2 mRNA. Axl2 mRNA was quantified by RT-qPCR in wild type cells or cells carrying *RNT1* deletion or mutation in its catalytic domain. Bar graphs show the mean value from 3 biological replicates shown as dots (**p < 0.01 by two-tailed unpaired t-test).

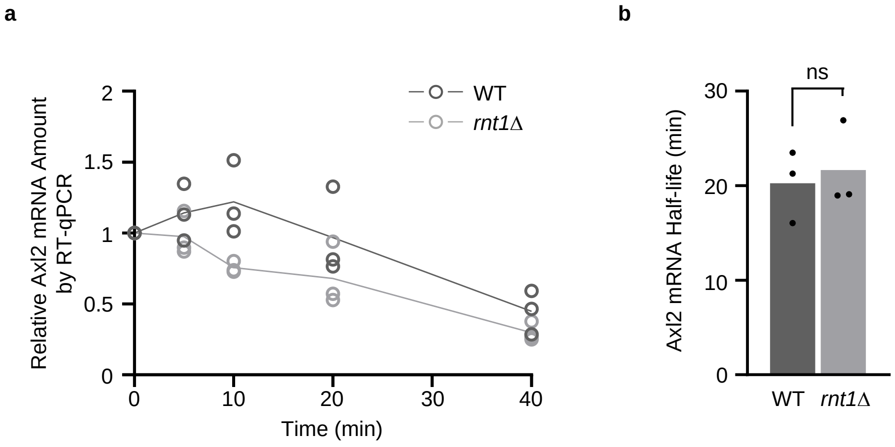

Supplementary Figure 8 (related to Fig. 4). Global Axl2 mRNA Stability is Unchanged in *rnt1*Δ

a, Impact of *RNT1* deletion on the stability of Axl2 mRNA after pression of transcription. RNA transcription was arrested in WT and *rnt1*Δ cells with thiolutin and the level of Axl2 mRNA was measured relative to Rpr1 RNA using RT-qPCR. The lines show the average values calculated from 3 biological replicates shown as circles.

b, Comparison of Axl2 mRNA decay rate in wild type cells and cells lacking *RNT1*. Decay rates were measured after 10 minutes of treatment with thiolutin. Shown are bar graphs representing the mean values measured from 3 biological replicates shown as dots with a non significant p-value of 0.7077 by two-tailed unpaired t-test.

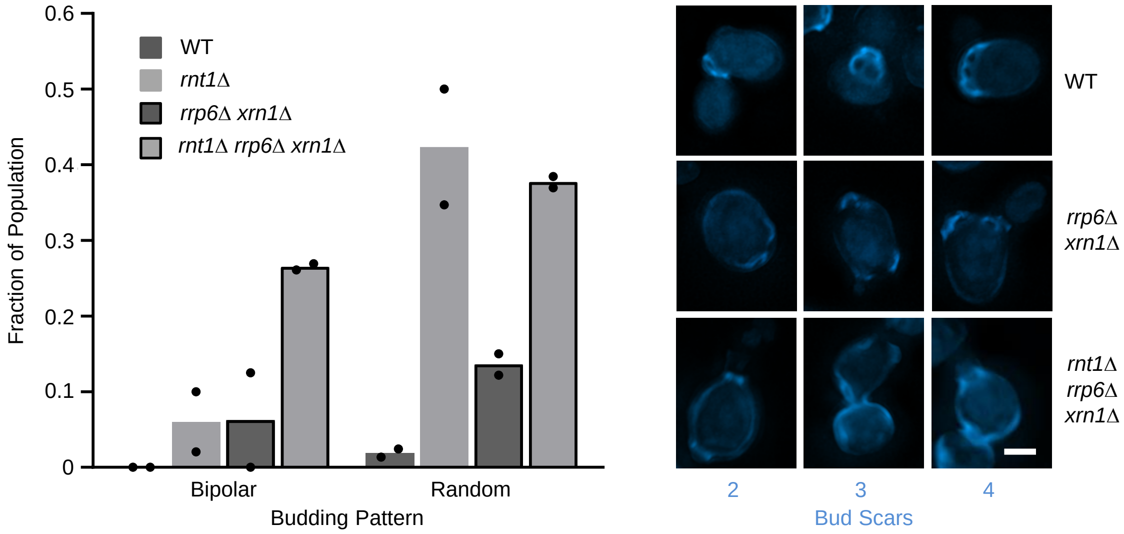

Supplementary Figure 9 (related to Fig. 4). Yeast Budding Pattern is Controlled by a Network of Nuclear Ribonucleases
The budding pattern was monitored as a function of scars location detected by calcofluor staining in WT and strains carrying 1-3 ribonuclease deletions. The bar graphs represent the average fraction of population showing non axial budding for 2 independent experiments shown as dots including 115 WT, 118 *rnt1Δ*, 86 *rrp6Δ xrn1Δ* and 72 *rnt1Δ rrp6Δ xrn1Δ* cells. Examples of scarring patterns as observed in cells carrying 2-4 scars are shown on the right. White bar equals 2 μm.

a **b**

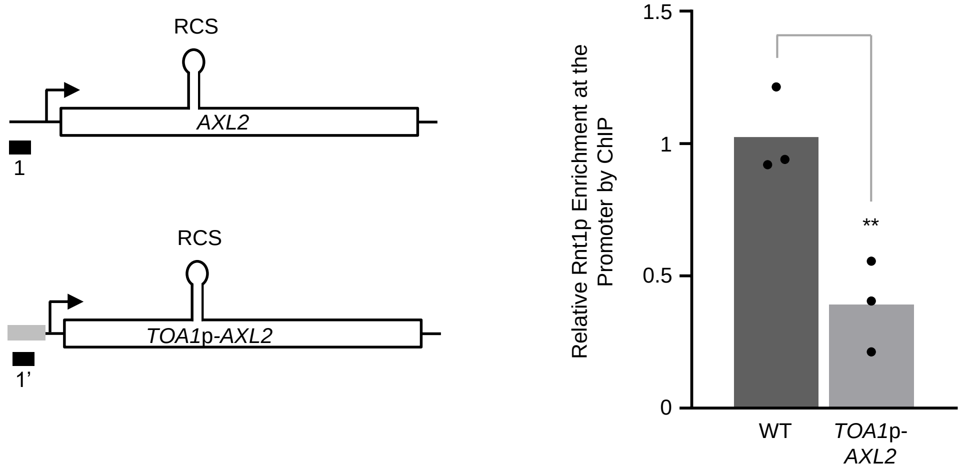

Supplementary Figure 10 (related to Fig. 5). Rnt1p Interacts with the Promoter of *AXL2*
a, Schematic representation of the *AXL2* locus depicting the position of the different fragments amplified by qPCR after chromatin immunoprecipitation. The position of Rnt1p cleavage site (RCS) is shown on top and the replaced promoter is depicted by a gray box. A probe set specific to the endogenous region (1) or to the replaced region was used (1’).
b, Promoter substitution reduces the association of Rnt1p to the chromatin upstream of *AXL2*. The Rnt1p association was examined in wild type cells (WT) or cells expressing *AXL2* from a heterologous promoter (*TOA*1p-*AXL2*) as described in Figure 5b with primers for amplicon 1 specific to the *AXL2* or *TOA1* promoter as required. The results from 3 biologically independent chromatin immunoprecipitations are presented as dots overlaid on the bar graphs showing the averages. Asterisks indicate significant difference between strains (**p < 0.01 by two-tailed unpaired t test).
