# Supplementary material for: Promoter-dependent nuclear RNA degradation ensures cell cycle-specific gene expression
Source: Commun Biol. 2019 Jun 17;2:211. doi: 10.1038/s42003-019-0441-3 (PMC6572803; doi:10.1038/s42003-019-0441-3)
Supplement: Supplementary file 3 — Description of additional supplementary items [file 42003_2019_441_MOESM3_ESM.docx]

Description of additional supplementary items

**Supplementary Data 1**

Excel document containing:

Table S1: Statistical analysis of the smFISH dataset

Table S2: Strains with genotypes used in this study

Table S3: Oligonucleotides sequences used in this study

**Supplementary Data 2**

Excel document containing the data used for the making of each figures and supplementary figures organized by one figure per sheet.

**Supplementary Data 3**

Excel document containing cell by cell data collected by smFISH organized by one strain per sheet.
